# Occurrence of Rotavirus A Genotypes and Other Enteric Pathogens in Diarrheic Suckling Piglets from Spanish Swine Farms

**DOI:** 10.3390/ani12030251

**Published:** 2022-01-20

**Authors:** Luis V. Monteagudo, Alfredo A. Benito, Sofía Lázaro-Gaspar, José L. Arnal, Desirée Martin-Jurado, Rut Menjon, Joaquín Quílez

**Affiliations:** 1 Department of Anatomy, Embryology and Genetics, Faculty of Veterinary Sciences, University of Zaragoza, 50013 Zaragoza, Spain; monteagu@unizar.es; 2 Agrifood Institute of Aragón (IA2), University of Zaragoza-CITA, 50013 Zaragoza, Spain; 3 EXOPOL S.L, Pol Rio Gállego D/14, San Mateo del Gállego, 50840 Zaragoza, Spain; abenito@exopol.com (A.A.B.); slazaro@exopol.com (S.L.-G.); jlarnal@exopol.com (J.L.A.); dmartin@exopol.com (D.M.-J.); 4 MSD Animal Health España, Carbajosa de la Sagrada, 37188 Salamanca, Spain; ruth.menjon.ruiz@merck.com; 5 Department of Animal Pathology, Faculty of Veterinary Sciences, University of Zaragoza, 50013 Zaragoza, Spain

**Keywords:** *Clostridium perfringens*, *Clostridioides difficile*, species A and C rotavirus, porcine epidemic diarrhea virus, pigs, occurrence, genetic diversity, Spain

## Abstract

**Simple Summary:**

Neonatal diarrhea is a major cause of economic losses in the swine industry worldwide and has significant impact in Spain, which is one of the biggest pork producers globally. Multiple infectious agents can contribute to this condition, with some viruses such as species A rotavirus (RVA) playing a major role. Studies on their occurrence and genetic diversity are essential for development of RVA vaccines. In this study, fecal samples from diarrheic suckling piglets originating from farms distributed throughout Spain were analyzed for RVA and four other common enteric pathogens using molecular methods. The individual prevalence was 89.4%, 64.4%, 44.9%, 33.7% and 4.4% for *Clostridium* *perfringens*, *Clostridioides* (formerly *Clostridium*) *difficile*, species A rotavirus, species C rotavirus and porcine epidemic diarrhea virus, respectively. Most specimens (96.9%) were positive for at least one of the target pathogens and concurrent infections were common. The molecular characterization of RVA positive specimens of specific genes used for genotyping revealed the extensive genetic diversity of RVA strains circulating in swine herds in Spain. Comparison with genotypes contained in the commercial vaccine available in Spain showed differences in the identity of the predominant RVA genotypes from diarrheic piglets in the sampled pig farms. These findings contribute to the surveillance of RVA strains circulating in swine herds in Spain and may help optimize target vaccine design.

**Abstract:**

Species A rotavirus (RVA) is a major viral pathogen causing diarrhea in suckling piglets. Studies on its genetic heterogeneity have implications for vaccine efficacy in the field. In this study, fecal samples (*n* = 866) from diarrheic piglets younger than 28 days were analyzed over a two-year period (2018–2019). Samples were submitted from 426 farms located in 36 provinces throughout Spain and were tested using real-time PCR (qPCR) and reverse transcription real-time PCR (RT-qPCR) for five enteric pathogens. The individual prevalence was 89.4%, 64.4%, 44.9%, 33.7% and 4.4% for *Clostridium* *perfringens*, *Clostridioides* (formerly *Clostridium*) *difficile*, species A rotavirus, species C rotavirus and porcine epidemic diarrhea virus, respectively. Most specimens (96.9%) were positive for at least one of the target pathogens, and more than 80% of samples harbored mixed infections. Nucleotide sequencing of 70 specimens positive for RVA revealed the presence of the VP7 genotypes G4, G9, G3, G5, G11 and the VP4 genotypes P7, P23, P6 and P13, with the combinations G4P7 and G9P23 being the most prevalent, and especially in the areas with the highest pig population. The study shows the extensive genetic diversity of RVA strains as well as discrepancies with the genotypes contained in the vaccine available in Spain, and multiple amino acid differences in antigenic epitopes of different G- and P- genotypes with the vaccine strains. Further investigations are needed to determine the efficacy of the vaccine to confer clinical protection against heterologous strains.

## 1. Introduction

Neonatal diarrhea is a major cause of economic losses in the swine industry worldwide. This multifactorial disease is estimated to account for up to 24% of the overall pre-weaning mortality in live-born piglets, and to reduce weight gain by up to 38 g per day [1,2]. The costs of neonatal porcine diarrhea for herds with a mortality of 10% can be as high as 134 € per sow per year [3]. Many factors can influence the occurrence of diarrhea in suckling piglets, including management procedures, immunity of piglets and a variety of infectious pathogens, with some viruses such as species A rotavirus (RVA) and coronaviruses (transmissible gastroenteritis virus (TGEV); porcine epidemic diarrhea virus (PEDV)), but also bacteria (enterotoxigenic *Escherichia coli* (ETEC), *Clostridium perfringens* types A and C, and *Clostridioides* (formerly *Clostridium*) *difficile*) being among the most common [4,5].

The genus rotavirus (RV) belongs to the family *Reoviridae* and is a nonenveloped and triple-layered virus. The rotavirus genome consists of 11 segments of double-stranded RNA (dsRNA) encoding six structural proteins (VP1–VP4, VP6, and VP7) and five or six non-structural proteins used in viral replication (NSP1–NSP5/6) [6]. Based on their distinct antigenicity and the sequence diversity of VP6, the inner capsid protein, rotaviruses have been classified into ten species (A–J). Of clinical and economic relevance for pigs are species A, B, and C [7]. Species A rotaviruses (RVA) are considered the most frequent viral agent involved in neonatal diarrhea in pigs and the only agent statistically associated with diarrhea in some studies [4,8]. Nevertheless, species C rotavirus (RVC) has been detected increasingly in swine in different countries and is currently recognized as a major single cause of gastroenteritis in neonatal piglets [9,10].

Rotaviruses are classified into G and P genotypes based on sequencing of the genes encoding two proteins forming the outer layer of the virus (VP7 and VP4). VP7 is a glycosylated protein and designates the G type, whereas VP4 is a protease-sensitive polypeptide and designates the P type [11]. At least 12 G (G1–G6, G8–G12, G26) and 17 P types (P1, P4–P11, P13, P14, P19, P23, P26, P27, P32, and P34) have been detected in RVA from swine based on this dual system, with G3–G5, G9 and G11 genotypes and P6, P7, P13, and P19 genotypes being common in Europe [7,11,12]. Nevertheless, a complete genome-based classification system comprising all the RV 11 dsRNA segments was proposed by the Rotavirus Classification Working Group (RCWG) for highly genetically diverse RVA strains [13]. A complete genome analysis of RVC strains has been done more recently and it confirmed the existence of at least 18G, 21P, 13I, 4R, 6C, 6M, 9A, 8N, 6T, 5E, and 4H genotypes for the genes VP7, VP4, VP6, VP1, VP2, VP3, NSP1, NSP2, NSP3, NSP4, and NSP5, respectively, in terrestrial mammals [14].

Regarding porcine coronaviruses, both transmissible gastroenteritis virus (TGEV) and porcine epidemic diarrhea virus (PEDV) can also cause diarrheal outbreaks with high morbidity and mortality in neonatal pigs. The clinical and economic impact of TGEV is low due to the emergence and rapid spread in the 1980s of the closely related porcine respiratory coronavirus (PRCV), which provides immunological cross-protection. In fact, most recent outbreaks in Europe have been related with PEDV [15,16]. A number of bacteria are also related to neonatal diarrhea in piglets. *Escherichia coli* strains are usually isolated from all submissions tested, but only some pathotypes are responsible for intestinal disease in pigs, mainly represented by enterotoxigenic (ETEC) and enteropathogenic (EPEC) *E. coli* [17]. Other significant enteric pathogens in suckling piglets are enterotoxigenic strains of *Clostridium* spp., including *C. perfringens* type C (producing α-and β-toxin) and *Clostridioides difficile* (producing enterotoxin A (TcdA) and/or cytotoxin B (TcdB)). The role of *C. perfringens* type A (producing α-toxin and ß2-toxin) as a cause of enteric diseases in pigs is less clear since disease has not been reproduced in inoculation studies in suckling pigs [18].

In Spain, the pork industry is experiencing expansion and continuous growth. In 2019, it accounted for 14% of total agricultural production and 39% of total livestock production, with a total of 31 million animals which represent the largest livestock category ahead of the cattle industry. Two regions located in the northeast of Spain concentrate the highest population of pigs: Aragón (26.2%) and Catalonia (25.3%) (https://www.mapa.gob.es/es/ganaderia/temas/produccion-y-mercados-ganaderos/sectores-ganaderos/porcino/, accessed on 18 January 2022). Spain also has the largest pig population in the EU and is the fourth largest producer of pork after China, the United States and Germany (https://ec.europa.eu/info/food-farming-fisheries/animals-and-animal-products/animal-products/pork_en, accessed on 18 January 2022). Despite this, studies on the impact of neonatal diarrhea and the occurrence of infectious agents involved in the etiology of this syndrome are limited in Spanish swine herds. Previous examinations have shown that RVA was the major viral pathogen and the only agent statistically correlated with the outcome of diarrhea, although other agents such as RVC, *C. perfringens* type A and *Clostridioides difficile* could also play a relevant role [5,8]. The genetic heterogeneity of porcine RVA is also poorly documented in Spain. Most isolates from a previous study with specimens from northeastern regions of the country were genotyped as G9P23 [19], but combinations G10P6, G12P8, G9P8 and G4P23 have also been found [11,20]. Lastly, there is a lack of information on the genomic similarity between circulating RVA strains and vaccine strains. Currently, the only vaccine available in Spain for the prevention of RVA diarrhea in young piglets has to be imported, mainly from USA (ProSystem Rota; Intervet Inc./Merck Animal Health, Madison, NJ, USA). The aim of this study was to investigate the frequency of different bacterial and viral pathogens involved in cases of neonatal diarrhea in swine farms throughout Spain. For this purpose, fecal samples were tested for five enteropathogens with a diagnostic panel for suckling piglet diarrhea: RVA, RVC, PEDV, *C. perfringens* and *Cl. difficile*. The genetic diversity of selected rotavirus strains from diarrheic piglets was also reported, and the genotypes compared to the strains contained in the swine RVA vaccine available in Spain.

## 2. Materials and Methods

### 2.1. Ethics Approval Statement

Fecal samples were collected from pigs by veterinary surgeons for diagnostic purposes after the permission of farm owners, with no specific permits being required by the authority for the feces collections. Animal care and use committee approval was not necessary for this study. Directive 2010/63/EU of the European Parliament on the protection of animals used for scientific purposes does not apply to non-experimental clinical veterinary practices.

### 2.2. Stool Samples

A total of 866 fecal samples from diarrheic domestic piglets (*Sus scrofa domestica*) younger than 28 days submitted to a veterinary diagnostic laboratory (Exopol S.L., San Mateo de Gállego, Spain) were used. Specimens were received in the form of fresh feces or sterile rectal cotton swabs over a period of two years (January 2018 to December 2019) from 426 farms located in 36 provinces throughout Spain. Multiple submissions corresponding to different diarrheic outbreaks on different dates were received from 126 out of 426 farms, usually with at least one month apart. The number of specimens received from each farm and outbreak ranged between 1 and 12 samples (mean, 1.21 ± 0.81). No data on the vaccination of sows against rotavirus or other enteropathogens in the studied herds were available in this study.

### 2.3. Nucleic Acid Extraction and qPCR or RT-qPCR

Nucleic acid isolation was performed with the commercial kit MagMAX™ Pathogen *RNA*/*DNA* (Thermo Fisher Scientific, Waltham, MA, USA) and an automated magnetic particle processor (KingFisher Flex; Thermo Fisher Scientific, Waltham, MA, USA), according to the manufacturer’s protocol and instructions. All specimens were tested by real-time PCR (qPCR) (*C. perfringens, Cl. difficile*) and reverse transcription real-time PCR (RT-qPCR) (RNA viruses) using commercial kits (EXOone qPCR kits, Exopol S.L., San Mateo de Gállego, Spain) for a diagnostic panel including group A rotavirus, group C rotavirus, porcine epidemic diarrhea virus (PEDV), *Clostridium perfringens* and *Clostridioides difficile*. These assays target the RVA non-structural protein NSP3, the major inner RVC capside protein VP6, PEDV nucleocapside (N) protein, *C. perfringens* alpha-toxin (CPA) gene, and *Cl. difficile* toxin B (TcdB) gene, respectively.

### 2.4. G and P Genotyping of RVA

**Figure 1:**
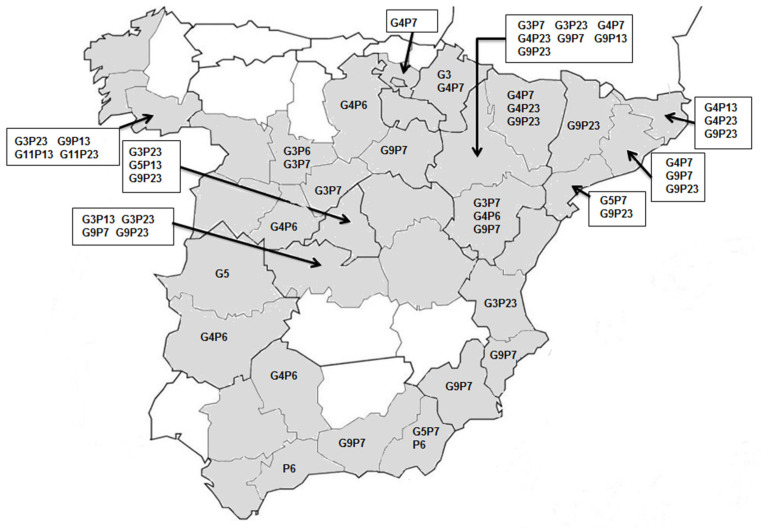
Map of Spain. Pig farms submitting samples for diagnosis of enteropathogens in this study were located in the shaded provinces. Some RVA strains from twenty-six provinces were genotyped at the VP7 and VP4 genes. The occurrence of genotype combinations is indicated.

A subset of 70 RVA strains were selected for nucleotide sequence analysis of VP7 and VP4 genes. These strains were obtained from fecal specimens belonging to different farms (1 sample/farm) in 26 provinces (Figure 1). PCR amplification of both genes was achieved using protocols described previously with some modifications. For G-typing, primers VP7F/VP7R [21] or Bov9Com5/Bov9Com3 [22] were used with annealing temperatures of 56 °C and 52 °C, respectively. For P-typing, primers Con2/Con3 [23] or Bov4com5/Bov4Com3 [22] were used with an annealing temperature of 50 °C. Each sample underwent amplification for VP7 and VP4 genes with the first of the above-mentioned primer pairs. Alternative primer pairs were used if no amplicon was observed or if the amplicon was not of the expected length. The PCR products were subjected to electrophoresis in 1.5% *w*/*v* agarose gels and visualized with a UV transilluminator. PCR products of positive samples were purified and sequenced in both directions with the forward and reverse primers used for amplification at STABvida laboratories (Caparica, Portugal).

### 2.5. Sequence Analysis

The RVA genotypes were determined according to the guidelines of the RCWG [13]. Alignment of the consensus sequences against each other and with reference sequences retrieved from GenBank was done using Clustal W and edited with BioEdit version 7.2.5 (https://bioedit.software.informer.com/versions/, accessed on 18 January 2022). Consensus sequences were compared with available rotavirus sequences in the NCBI databases using the Basic Local Alignment Search Tool (http://blast.ncbi.nlm.nih.gov/Blast.cgi, accessed on 18 January 2022). Neighbor-joining phylogenetic trees were constructed for VP7 and VP4 sequences by means of the MEGA software (https://www.megasoftware.net/, accessed on 18 January 2022). The robustness of branching patterns was tested by 1000 bootstrap replicates. Tree drawing was performed online by means of the iTOL v5 tool [24]. The amino acid sequence of the neutralization epitopes described in VP7 and VP4 was deduced for all Spanish pig RVA strains and compared with the amino acid composition of the same antigenic epitopes in the vaccine strains Gottfried G4P6, OSU G5P7, and A2 G9P7 [12]. Translation was performed by means of the EMBL-EBI translation tool [25].

### 2.6. Nucleotide Sequence Accession Numbers

Nucleotide sequences of both VP7 and VP4 genes generated in this study were deposited in the GenBank database under accession numbers MZ643273 to MZ643406.

### 2.7. Statistical Analysis

Data were analyzed using SPSS version 26 (SPSS Inc., Chicago, IL, USA, 2019) software. Chi-square tests were used to evaluate possible significant differences in the occurrence of the target enteropathogens in fecal specimens and to analyze seasonal and yearly variations. Values of *p <* 0.05 were considered statistically significant.

## 3. Result

### 3.1. Detection of Enteropathogens

A summary of the number of specimens and farms testing positive for any of the five enteropathogens examined is indicated in Table 1. A total of 389 stool samples (44.9%; 95% CI: 42–48%) from 250 farms (58.7%; 95% CI: 54–63%) were reported to be positive for species A rotavirus. This pathogen was significantly more prevalent than species C rotavirus, which was found in 292 stool specimens (33.7%; 95% CI: 31–37%) from 188 farms (44.1%; 95% CI: 39–49%). Nevertheless, the most frequently detected and widespread agents were *Clostridium* and *Clostridioides* species. Most specimens (89.4%; 95% CI: 87–91%) and farms (93.9%; 95% CI: 91–96%) tested positive for *C. perfringens*, followed by *Cl. difficile* which was detected in 558 samples (64.4%; 95% CI: 61–68%) from 320 farms (75.1%; 95% CI: 71–79%). The least common of the five target enteropathogens was PEDV. This agent was found in 38 specimens (4.4%; 95% CI: 3–6%) from 30 farms (7%; 95% CI: 5–10%). Statistical analyses revealed significant differences among the prevalence of the different enteropathogens (*p* < 0.0001). A total of 142 samples (16.4%) were positive for only one of these pathogens. The coinfection with at least two pathogens was detected in more than 80% fecal samples (*n* = 697), with the combination of *C. perfringens* + *Cl. difficile* alone or mixed with RVA or RVC being reported in more than a half of the fecal specimens (*n* = 453). Only 27 stool specimens (3.1%) tested negative for all the pathogens. All the target pathogens were detected throughout the year with spatio-temporal fluctuations but without evidence of seasonality. Nevertheless, the annual prevalence significantly decreased from 2018 to 2019 for RVA (49.3% to 41.9%), RVC (37.7% to 30.9%) and *Cl. difficile* (68.8% to 61.4%) (*p* < 0.05).

The mean prevalence of RVA per month in the two-year period ranged from 32.3% (October) to 54% (July). The raw data of detection of enteric pathogens can be found in the Supplementary Table S1.

### 3.2. Rotavirus Genotypes

A total of 64 of the 70 specimens selected for RVA genotyping were successfully characterized at both the VP7 (G) and VP4 (P) genes. Five different G genotypes were found, with G4 (n: 26) and G9 (n: 23) being the most prevalent, followed by G3 (n: 12), G5 (n: 4) and G11 (n: 2). Among the four P genotypes identified, P7 (n: 28) and P23 (n: 23) were dominant, and the remaining were P6 (n: 10) and P13 (n: 6) (Table 2). Repeated attempts to sequence six specimens for either the G (n: 3) or the P (n: 3) genotype were unsuccessful and these specimens were considered un-typeable. Fifteen G-P genotype combinations were identified, with G4P7 and G9P23 being the most common. Eight combinations were reported in only one or two specimens each. Genotypes were distributed throughout Spain, although the two most common combinations (G4P7 and G9P23) were mostly distributed on the two northeastern regions (Aragón, Catalonia) while other common combinations (G9P7 and G4P6) were spread over many regions. The occurrence of all genotype combinations is shown in Figure 1.

Nucleotide sequence comparison of the different genotypes revealed a large diversity. The highest number of single nucleotide polymorphisms (SNPs) of the VP7 gene was found at the G4 genotype. Alignments identified up to 343 SNPs in 981 bp of sequence (0.349 SNPs/nt). Nevertheless, to prevent any bias related to the difference in the number of samples, an index of 343/981/26 (SNPs/nt/sample) was calculated (0.0134). Genotypes P6 and P13 provided the highest index at the VP4 gene (0.0297 and 0.0583 SNPs/nt/sample, respectively) (Table 3).

**Figure 2:**
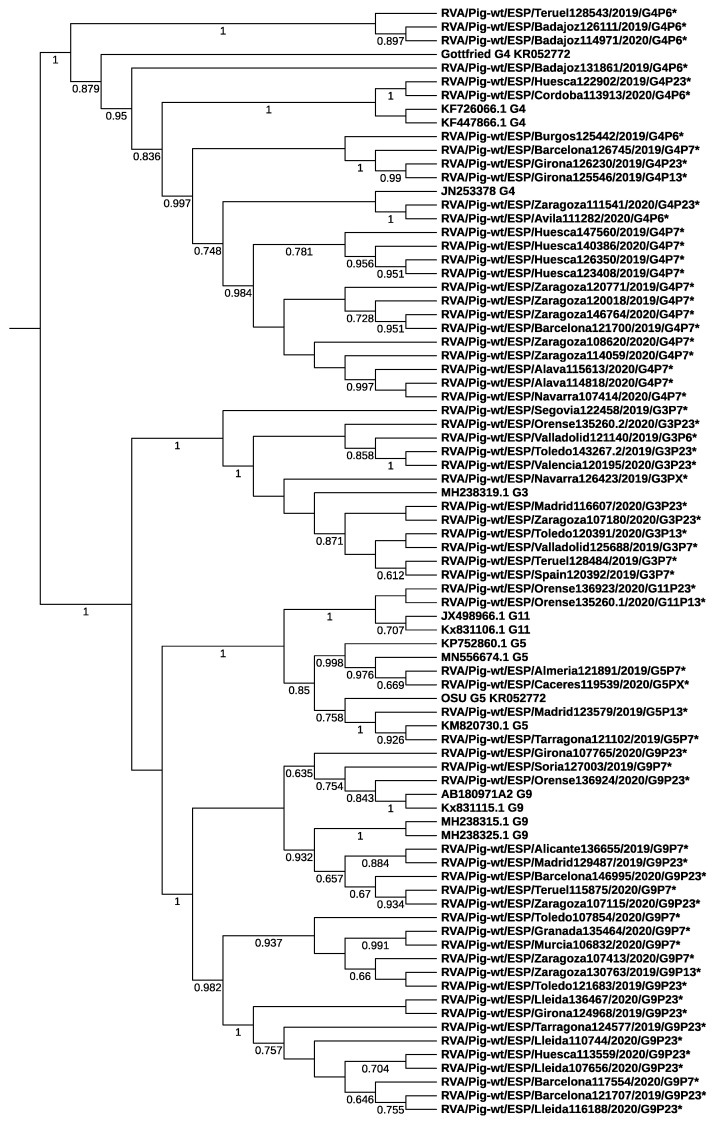
Phylogenetic relationships of G genotypes of RVA strains examined in the current study (marked with *) to representative strains of different G genotypes and vaccine strains RVA/Pig-tc/USA/LS00007_Gottfried/1975/G4P6 (KR052751), RVA/Pig-tc/USA/LS00005_OSU/1975/G5P9 [7] (KR052772) and RVA/Pig-tc/USA/A2/198x/G9P9 [7] (AB180971). Neighbor joining was calculated following the Kimura two-parameter method and branches support was estimated by bootstrap with 1000 replicates (only support values over 0.6 in a 0–1 scale are shown). The genotype nomenclature follows the recommendations of the RCWG.

**Figure 3:**
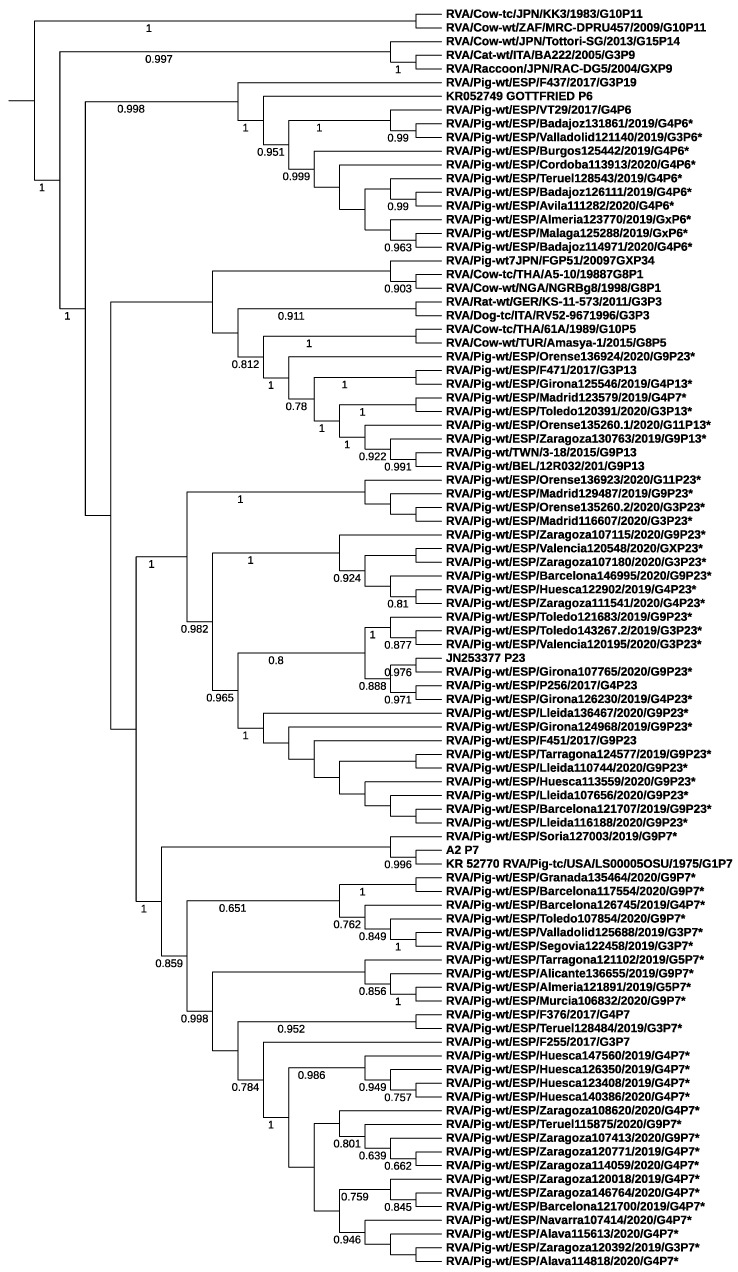
Phylogenetic relationships of P genotypes of RVA strains examined in the current study (marked with *) to representative strains of different P genotypes and vaccine strains RVA/Pig-tc/USA/LS00007_Gottfried/1975/G4P6 (KR052749), RVA/Pig-tc/USA/LS00005_OSU/1975/G5P9 [7] (KR052770) and RVA/Pig-tc/USA/A2/198x/G9P9 [7] (AB180977). Neighbor joining was calculated following the Kimura two-parameter method and branches support was estimated by bootstrap with 1000 replicates (only support values over 0.6 in a 0–1 scale are shown). The genotype nomenclature follows the recommendations of the RCWG.

Phylogenetic analysis based on the VP7 and VP4 genes showed that RVA strains belonging to different genotypes segregated into distinct branches and clustered with porcine strains from Spain and other countries (Figure 2 and Figure 3). No clear cluster of samples collected from farms in nearby regions was observed, indicating no strong phylogeographic structure. Strains from this study were related to RVA strains collected in 2017 from pig farms in northeastern Spain by Vidal et al. (>92% nucleotide similarity) [19]. For example, the current G3 strain 120195 and G9 strain 136655 clustered closely to previous strains with GenBank accession number MH238319.1 (92% nucleotide similarity) and MH238315.1 (93%), respectively. At the VP4 gene, the sequence homology between the current P7 strain 128484 and the previous porcine strain F255 (MH238272) was 93%.

### 3.3. Differences among Field Isolates and Vaccine Strains

The nucleotide and corresponding amino acid sequence identities of porcine RVA strains to each other is illustrated in Supplementary Tables S2–S11. Comparison of major genotypes of this study revealed 81.9–99.9% nucleotide identity for the G(4) genotype, 88.5–99.6% for the G(9) genotype, 82.5–99.8% for P7 genotype and 82.6–99.7% for the P23 genotype. Comparison with prototype strains contained in the vaccine available in Spain (ProSystem Rota; Intervet Inc./Merck Animal Health) showed nucleotide similarity with the porcine Gottfried strain G(4) (82.8–87.2%), A2 strain G(9) (91.4–96.8%), OSU strain G(5) (84.9–87.9%), Gottfried strain P6 (80.9–83.3%), OSU strain P7 (86–88.4%) and A2 strain P7 (83.2–85.6%).

An amino acid analysis of the neutralization epitopes on VP7 and VP4 genes of Spanish porcine RVA strains is indicated in Supplementary Tables S12 and S13, respectively. Three neutralizing domains have been described in the literature for the VP7 glycoprotein, namely, 7-1a, 7-1b and 7-2 [26]. For the VP4 protein, four putative neutralization regions (8-1, 8-2, 8-3 and 8-4) have been defined [26]. For the VP7 glycoprotein, 10 out of 29 residues were conserved among all Spanish RVA strains, including seven residues in 7-1a (98, 99, 100, 104, 123, 125, 129), one residue in 7-1b (211) and two residues in 7-2 (143, 264). The number of residues conserved among all isolates was lower for VP4 (6 out of 25), including three residues in 8-1 (100, 188, 193), one residue in 8-2 (180) and two residues in 8-3 (131, 132). Comparison of amino acid residues with corresponding residues of the vaccine strains revealed that all but four Spanish strains from sampled piglets had some mutations in any of the three antigenic regions of the VP7 protein, with more than half of isolates (41/67) showing differences in at least 4 amino acid residues, and numerous strains exhibiting 6-7 mutations. A higher number of amino acid changes was seen in VP4, with all Spanish strains exhibiting at least one mutation in this protein and 39 strains showing 8-12 amino acid differences with vaccine sequences. All P6 strains differed in 10–12 amino acid residues from the Gottfried P6 strain. The number of amino acid differences between P7 strains and vaccine strains OSU P7 and A2 P7 was lower (1–3).

## 4. Discussion

Molecular tools have contributed to unraveling the complexity of infectious causes of neonatal diarrhea in pigs. In this study, we used a real-time PCR approach to investigate the occurrence of five enteropathogens in diarrheic specimens from piglets and 97% of samples tested positive for at least one of them, which indicates that these viral and bacterial agents are commonly involved in the etiology of infectious pig diarrhea in Spain. It is worth mentioning that a majority of cases (>80%) corresponded to mixed infections with different combinations of the target agents, a finding which has also been highlighted in previous studies in Spanish herds [5,8]. Diarrhea in a herd may be due to a single agent, but the finding of multiple concurrent agents is being reported increasingly in clinics, frequently with complex interactions that can result in synergistic or additive effects leading to more severe and prolonged diarrhea [27,28]. Enterotoxigenic *E. coli* (ETEC) strains were not included in the diagnostic panel in the current study due to earlier studies indicating that their prevalence is very low in neonatal piglet diarrhea in Spanish herds. This finding has also been reported in other European countries and has been linked to routine vaccination of sows [5,8,29,30].

Some studies have reported a seasonal pattern of infections with enteric pathogens associated with piglet diarrhea. In Canada, Chan et al. [31] found a significant trend for cases submitted in the winter to be diagnosed with *C. perfringens* type A, enterotoxigenic *E. coli*, rotavirus and *Cystoisospora suis*. A higher prevalence of rotavirus infections was reported during the summer and winter seasons in India [32]. In contrast, no evidence of seasonality in the presence of rotavirus infection in swine was documented in other studies in Canada, the United States and elsewhere [7,33,34]. In this study, both rotavirus and *C. perfringens/Cl. difficile* were consistently found over all months sampled with no seasonal trend, although the prevalence of both rotavirus and *Cl. difficile* significantly decreased from 2018 to 2019.

*C. perfringens* was by far the most prevalent enteric pathogen with more than 89% of samples and 93% of farms testing positive. The involvement of this bacterium in the etiology of diarrheic outbreaks in this study was uncertain since the PCR assay used targeted the alpha-toxin gene, which is produced by all toxinotypes. *C. perfringens* is classified into five toxinotypes (A through E) based on the production of four major toxins (alpha-, beta-, epsilon- and iota-toxins). Some toxinotypes (*C. perfringens* type C) are a well-established cause of enteritis in suckling piglets, although they appear to play a minor role currently in the development of disease due to routine vaccination of the breeding stock [4,30]. The role of other toxinotypes commonly found in piglets such as *C. perfringens* type A containing the beta2 toxin gene (CPA cpb2) remains unclear as they are considered part of the normal intestinal microbioma [4,27,30].

*Cl. difficile* was the second most prevalent pathogen in this study. It has been considered a potential enteric pathogen in pigs during the first week of life [18], although the relationship between diarrheal outbreaks and the detection of toxigenic *Cl. difficile* is also controversial. *Cl. difficile* dosage and piglet-age appear to be important factors that influence the severity of clinical signs and histological lesions [35], but some studies have reported that its prevalence is similar in both healthy and diarrheic animals [4,8,30]. In the current study, more than 74% of cases associated with *Cl. difficile* involved mixed infections with RVA and/or RVC, and cases attributed to only this bacterium were scarce (*n* = 13). In Spain, both *C. perfringens* type A and *Cl. difficile* have been isolated from most pig farms while *C. perfringens* type C was detected at a low prevalence [8]. Mesonero-Escuredo et al. [5] found *C.* *perfringens* type A in most fecal specimens (89.9%) but *C. perfringens* type C was not found. *Cl. difficile* contamination is also well established within the pig farm environment in northern Spain where more than 40% of farms presented at least one positive sample, although only a low proportion of farms (7.4%) tested positive in pig feces [36].

Regarding the viral etiology of neonatal diarrhea, RVA is documented to have important clinical significance in piglets [4]. In the United States, RVA was demonstrated in 63% of diarrheic samples submitted to the University of Minnesota although earlier studies detected much lower prevalence in swine farms from Ohio (9.4%) [9,33]. Studies in European pig farms have also shown varying values of prevalence, ranging from 4.2% to 19.9% according to a study by Midgley et al. [11] in four countries (Hungary, Denmark, Spain, Slovenia), or only 0.9% reported by Zhou et al. [37] in pigs from five European countries (Austria, Germany, Hungary, Spain, Sweden). Another significant viral contributor to the burden of diarrheal disease in nursing pigs is RVC, which is being increasingly detected in swine in different countries [7]. In the USA and Canada, this virus was reported in 37% of specimens from piglets younger than 20 days, but the frequency increased to 76.1% in another study in the USA [38,39]. The prevalence of RVC in most European pig farms is poorly documented. One study in Italian pig farms reported a prevalence of 31.3% in piglets with gastroenteritis and another study detected RVC in 22.3% nursing piglets in the Czech Republic [10,40]. In contrast, the study by Zhou et al. [37] in pig farms from five European countries revealed the presence of RVC in a small percentage of enteric infections (3%).

In this study, RVA was found in 45% of diarrheic piglets from 59% of farms. The prevalence and distribution of RVC was also significant, with more than 34% of fecal specimens and 44% of farms testing positive. Additionally, evidence was obtained for the simultaneous circulation of both viruses in more than 20% of the herds. Previous studies in Spain have shown that RVA infections were significantly more frequent in pigs with diarrhea (26.5%) than in asymptomatic animals (6.5%) [20]. In north-eastern Spain, RVA was identified in 61.4% of diarrheic piglets and was the only enteropathogen statistically correlated with the cases of diarrhea [8]; these authors also identified RVC in 33.6% of diarrheic piglets. Another study in piglets from Spanish farms with neonatal recurrent diarrhea reported that 43.1% of submissions were positive for RVA [5]. In contrast, RVA was not detected in 83 specimens from healthy and diarrheic pigs of different ages in 26 farms in Spain while 13.9% specimens were positive for RVC [37].

The results of this study showed that PEDV plays a secondary role in the etiology of diarrhea in neonatal swine in Spanish farms. It was by far the less common of the target pathogens investigated since less than 5% of specimens and 7% farms were positive for this coronavirus. PEDV can cause watery acute diarrhea in swine of all ages, but the severity of infection is higher in piglets younger than one week [15]. The virus was first recognized for the first time in Europe in the 1970s, although a major concern reappeared in 2014 in several European countries, where the virus caused high piglet mortality and significant economic losses in Germany [41], France [42], Belgium [43], Ukraine [44], Austria [45], Portugal [46] and the Netherlands [47]. In Spain, sporadic outbreaks have been reported since 2014, suggesting a re-emergence similar to that described in these countries. The percentage of farms testing positive for PEDV in piglets during the first week of life has been reported to range from 3.7% to 12.9% [5,8], but this virus was seldom found in samples from diarrheic piglets less than seven days old routinely submitted to Spanish veterinary diagnostic laboratories in 2017 [48]. However, a recent study on 106 Spanish pig farms investigated between 2017 and 2019 revealed that PEDV was the only coronavirus detected in 38.7% of viral suspected diarrhea outbreaks, although most of them occurred in fattening pigs or post-weaning growing pigs, and to a lesser extent in diarrhea affecting nursing piglets, which the authors related to the maternal immunity in the sows or high biosecurity level in farrowing facilities [49].

The current study demonstrates a wide range of RVA genotypes circulating within the sampled Spanish pig farms. Additionally, isolates showed great genetic diversity as demonstrated by the high frequency of single nucleotide polymorphisms detected, especially at some genotypes of both the VP7 and VP4 genes. A total of 15 genotype combinations of five G genotypes (G3, G4, G5, G9, G11) and four P genotypes (P6, P7, P13, P23) were identified from 70 RVA strains. Nevertheless, two G genotypes (G4 and G9) and two P genotypes (P7, P23) accounted for more than 70% of specimens, with combinations G4P7 and G9P23 accounting for almost 40% of specimens. A large diversity of RVA genotypes was also reported in a recent study with samples from piglet diarrheic outbreaks in northeastern regions of Spain, where nine combinations were found from a total of 24 specimens analyzed [19]. Most isolates in the latter study (58%) were also genotyped as G9P23 but combination G4P7 was identified in a single specimen. In contrast, these combinations were not reported in a previous study also in northeastern Spain, where Halaihel et al. [20] identified G10P6, G12P8, G9P8 and G4P23 over a total of 30 specimens analyzed. These findings suggest a change of predominant genotypes over a period of time in the same geographical area as indicated previously in other areas or Europe [12]. It is worth mentioning that the two most frequent combinations found in the present study, G4P7 and G9P23, were mainly located in the two northeastern regions of Spain (Aragón and Catalonia) where more than half of pig production is concentrated. The predominance of the combination G9P23 and especially the genotype G9 has also been reported in Germany, which is another important European pig producer [50]; these authors suggested that the emergence of RVA strains with genotype G9 might have been accelerated by the large swine populations. In contrast, the combination G4P7 seems to be rare in Europe since it has been detected sporadically only in southern Ireland, the United Kingdom and Belgium [51,52,53].

Previous studies in Europe have shown a large diversity and spatiotemporal differences in the distribution of RVA G- and P- genotypes circulating in swine. The genetic heterogeneity of porcine RVAs is higher than that seen in cattle and comparable to that reported in humans, despite the fact that many fewer strains have been characterized in swine [7,12]. Midgley et al. [11] analyzed 1101 fecal samples from pigs in four countries (Denmark, Hungary, Slovenia, Spain) and identified 21 G-P combinations, although no single combination was predominant across Europe. Papp et al. [12] reported genotypes G5, G4 and G3 in association with P6 or P13 to be the most common in Europe, but differences between countries and fluctuations over time in the distribution of dominant RVA strains were identified. With regard to the distribution of dominant G-genotypes in this study, G4 was also the most prevalent in Germany and Denmark and is important in Poland, Slovenia, Italy, Belgium and the United Kingdom. G9 is the G-genotype most often found in pigs in Germany and Italy and was among the dominant genotypes in Belgium and the United Kingdom, but its prevalence was very low in Denmark and was not detected in Poland or Slovenia [11,50,52,53,54,55,56]. Concerning the P-type specificities, the dominant genotype P7 in this study is considered the most common VP4 porcine RVA type globally, although it is more prevalent in the Americas and Asia than in Europe [12]. Genotype P7 was predominant in Belgium and Poland and the third most common P-genotype in Slovenia and the UK, but its prevalence was very low in southern Ireland and Germany. In contrast, P23 was the most important P-genotype in Germany and Italy and it was reported in a few specimens in Belgium (11%) and the UK (2%), but it was not found in Poland, Slovenia or the Irish Republic [50,51,52,53,54,55,56].

The continuous monitoring of dominant porcine RVA strains is crucial for the design of vaccines, since a cause of ineffective vaccination could be infection by strains that are different antigenically from the prototype vaccine strains [57]. In humans, clinical trials have demonstrated immunological cross-reactivity and cross-protection of some rotavirus vaccines against disease induced by non-vaccine genotypes [58]. However, the performance of RVA vaccines for pigs against partially or fully heterotypic strains is unknown. Currently, the only RVA vaccine available for swine in Spain (ProSystem Rota; Intervet Inc./Merck Animal Health) has to be imported from the United States and is licensed for use in pregnant sows or piglets. This vaccine, which is increasingly used in Spanish pig farms, consists of porcine RVA strains Gottfried (G4P6), OSU (G5P7) and A2 (G9P7) which have been modified a long time ago [12]. Moise et al. [59] used computational tools to analyze the potential efficacy of this vaccine and demonstrated that T cell epitope cross-conservation between the vaccine and RVA porcine strains circulating in the United States is genotype-specific and limited to homologous strains. However, more than 200,000 sows are vaccinated every year in Spain with this vaccine (personal communication of R. Menjon) and no pharmacovigilance events suggesting a possible lack of efficacy have been reported to the Spanish Agency of Medicines and Medical Devices (available at https://www.aemps.gob.es/acciones-informativas-medicamentos-veterinarios/?cat=291, accessed on 18 January 2022).

The lack of information about rotavirus vaccination in the studied swine herds constitutes a limitation of this study. Nevertheless, our results reveal discrepancies between the predominant G- and P- genotypes of porcine RVAs circulating in the sampled swine farms and the vaccine strains. Namely, less than 25% of isolates were allocated to the vaccine genotype combinations in the vaccine strains, and the genetic distance between Spanish pig RVA strains and vaccine strains was relatively high. Comparisons of both G and P genotypes with historic strains OSU and Gottfried revealed <88.4% nucleotide identity, and <86% nucleotide similarity was seen with the P genotype of the A2 vaccine strain. The discrepancy was further supported by analysis of amino acid mutations of the main antigenic regions of VP7 and VP4 genes, which showed that only one third or less of the neutralization epitopes (10/29 and 6/25, respectively) were conserved between the field and vaccine strains with a notable high number of amino acid mutations (10–12) seen between our P6 isolates and the Gottfried P6 strain. Comparison with RVA strains collected in 2017 by Vidal et al. [19] from pig farms in northeastern Spain showed changes in numerous amino acid residues of the antigenic regions of both proteins.

## 5. Conclusions

The current study demonstrates that *Clostridium perfringens*, *Clostridioides difficile*, RVA and RVC are prevalent microorganisms in diarrheic suckling pigs in Spain, with concurrent infections being common and PEDV playing a minor role. The study also highlights the genetic diversity of RVA genotypes circulating in the sampled pig farms, with two predominant genotype combinations (G4P7 and G9P23) and a high frequency of SNPs in the genotypes identified. Comparisons between pig RVA strains circulating in the sampled pig farms and vaccine strains reveal discrepancies in the identity of some G- and P- genotypes and amino acid mutations in antigenic epitopes. Further epidemiological investigations are needed to determine the RVA strains circulating in vaccinated pig herds and the efficacy of the vaccine to confer clinical protection against heterologous strains.

## Figures and Tables

**Table 1:** Enteropathogens identified in fecal specimens from piglets younger than 28 days submitted to a veterinary diagnostic laboratory in Spain [number positive (%)].

Enteropathogen	No. Piglets (*n* = 866)	No. Farms (*n* = 426)
*C. perfringens*	99 (11.4)	69 (16.2)
*Cl. difficile*	13 (1.5)	9 (2.1)
RVA	25 (2.9)	22 (5.2)
RVC	3 (0.3)	2 (0.5)
PEDV	2 (0.2)	1 (0.2)
*C. perfringens + Cl. difficile*	121 (13.9)	93 (21.8)
*C. perfringens* + RVA	82 (9.5)	72 (16.9)
*C. perfringens* + RVC	31 (3.6)	27 (6.3)
*C. perfringens* + PEDV	7 (0.8)	6 (1.4)
*C. perfringens + Cl. difficile* + RVA	172 (19.9)	129 (30.3)
*C. perfringens + Cl. difficile* + RVC	160 (18.5)	121 (28.4)
*C. perfringens + Cl. difficile* + PEDV	7 (0.8)	7 (1.6)
*C. perfringens* + RVA + RVC	14 (1.6)	13 (3)
*C. perfringens* + RVA + PEDV	6 (0.7)	5 (1.2)
*C. perfringens* + RVC + PEDV	1 (0.1)	1 (0.2)
*C. perfringens* + *Cl. difficile* + RVA + RVC	65 (7.5)	54 (12.7)
*C. perfringens + Cl. difficile* + RVA + PEDV	4 (0.5)	4 (0.9)
*C. perfringens + Cl. difficile* + RVC + PEDV	3 (0.3)	2 (0.5)
*C. perfringens* + RVA + RVC + PEDV	2 (0.2)	2 (0.5)
*Cl. difficile* + RVA	6 (0.7)	3 (0.7)
*Cl. difficile* + RVC	3 (0.3)	3 (0.7)
*Cl. difficile* + RVA + RVC	4 (0.5)	3 (0.7)
RVA + RVC	3 (0.3)	3 (0.7)
RVA + PEDV	3 (0.3)	3 (0.7)
RVA + RVC + PEDV	3 (0.3)	3 (0.7)
No detection	27 (3.1)	18 (4.2)

**Table 2:** Combinations of G and P genotypes in 70 specimens testing positive for species A rotavirus. Samples were randomly selected from diarrheic piglets at different farms in Spain (1 sample/farm).

P—G Type	No. of Samples
G4P [7]	14
G9P [23]	13
G9P [7]	8
G4P [6]	7
G3P [7]	4
G3P [23]	5
G4P [23]	3
G3P [13]	1
G9P [13]	2
G5P [7]	2
G3P [6]	1
G4P [13]	1
G5P [13]	1
G11P [13]	1
G11P [23]	1
G3P [X]	1
G4P [X]	1
G5P [X]	1
XP [6]	2
XP [23]	1
Total	70

X: not typeable.

**Table 3:** Maximum number of single nucleotide polymorphisms (SNPs) identified after alignment of nucleotide sequences of the different genotypes identified in this study. The index was calculated using the sequence with the largest number of nucleotides and the number of samples identified for each genotype.

Genotype	No. Maximum of SNPs/nt/Sample	Index
G4	343/981/26	0.0134
G9	274/981/23	0.0121
G3	157/981/11	0.0145
G5	202/981/4	0.0515
G11	40/979/2	0.0204
P [7]	270/838/28	0.0115
P [23]	290/838/23	0.0150
P [6]	249/838/10	0.0297
P [13]	293/838/6	0.0583

## Data Availability

Nucleotide sequences of both VP7 and VP4 genes generated in this study were deposited in the GenBank database under accession numbers MZ643273 to MZ643406. The remaining data presented in this study are contained within this article.

## Supplementary Materials

The following are available online at https://www.mdpi.com/2076-2615/12/3/251/s1, Table S1: Raw data of detection of enteric pathogens. Tables S2–S10: Nucleotide similarity (%) among RVA strains of the different genotypes identified in the current study and the prototype strains contained in the vaccine available in Spain. Similarity among the amino acids coded by the same sequences is also indicated. Table S11: Identity of RVA strains according to the nomenclature proposed by the RCWG. Tables S12 and S13: Amino acid residues defining neutralization domains of genotypes of the glycoprotein VP7 (Table S12) and VP4 (Table S13). Amino acid mutations between Spanish pig strains from the present study and prototype vaccine strains.
