# Supplementary material for: Occurrence of Rotavirus A Genotypes and Other Enteric Pathogens in Diarrheic Suckling Piglets from Spanish Swine Farms
Source: Animals (Basel). 2022 Jan 20;12(3):251. doi: 10.3390/ani12030251 (PMC8833434; doi:10.3390/ani12030251)
Supplement: Supplementary file 1 [file animals-12-00251-s001.zip › suppl Table S1.pdf]

Table S1: Raw data of detection of enteric pathogens.

Date	Farm ID	*C. perfringens*	*Cl. difficile*	RVA	RVC	PEDV
2018/1/3	EXP819	+	+	-	+	-
2018/1/5	EXP319	+	+	-	+	-
2018/1/11	EXP254	+	+	+	-	-
2018/1/11	EXP792	+	+	+	+	-
2018/1/11	EXP792	+	+	-	+	-
2018/1/16	EXP091	+	-	+	+	-
2018/1/16	EXP126	+	-	+	-	-
2018/1/16	EXP127	+	+	+	+	-
2018/1/16	EXP261	+	-	+	-	-
2018/1/16	EXP262	+	-	-	+	-
2018/1/17	EXP087	+	+	+	+	-
2018/1/18	EXP309	+	+	+	+	-
2018/1/18	EXP252	+	+	+	-	-
2018/1/19	EXP819	+	+	+	-	-
2018/1/23	EXP260	+	+	+	-	-
2018/1/24	EXP326	+	-	+	-	-
2018/1/24	EXP138	+	+	+	-	-
2018/1/24	EXP239	+	-	-	-	-
2018/1/24	EXP255	+	-	+	+	-
2018/1/25	EXP015	+	-	+	-	-
2018/1/25	EXP155	+	-	-	+	-
2018/1/25	EXP155	+	-	-	-	-
2018/1/25	EXP155	+	-	-	-	-
2018/1/25	EXP250	+	-	-	-	-
2018/1/25	EXP800	+	+	+	+	-
2018/1/26	EXP129	+	+	+	-	-
2018/1/29	EXP796	+	+	-	+	-
2018/1/30	EXP149	+	+	-	+	-
2018/1/30	EXP761	+	+	+	-	-
2018/1/31	EXP198	+	-	+	-	-
2018/1/31	EXP331	+	+	+	-	-
2018/1/31	EXP332	+	+	+	-	-
2018/2/1	EXP795	+	+	-	+	-
2018/2/2	EXP305	+	+	+	-	-
2018/2/7	EXP135	+	-	+	-	-
2018/2/7	EXP337	+	+	+	-	-
2018/2/7	EXP222	+	+	-	-	-
2018/2/7	EXP260	+	+	+	-	-
2018/2/8	EXP244	+	+	-	+	-
2018/2/9	EXP050	+	-	-	-	-
2018/2/14	EXP135	+	+	-	-	-
2018/2/14	EXP344	+	+	+	-	-
2018/2/14	EXP198	+	+	+	+	-
2018/2/14	EXP316	+	+	-	-	-
2018/2/14	EXP345	+	+	+	+	-
2018/2/14	EXP346	+	+	+	-	-
2018/2/15	EXP187	+	+	-	+	-
2018/2/15	EXP347	+	-	-	-	+
2018/2/16	EXP843	+	+	+	-	-
2018/2/16	EXP349	+	-	-	+	-
2018/2/20	EXP042	+	-	+	+	-
2018/2/20	EXP149	+	+	-	+	-
2018/2/20	EXP352	+	+	-	+	-
2018/2/20	EXP352	+	+	+	-	-

2018/2/20	EXP247	+	-	-	+	-
2018/2/20	EXP764	+	-	+	-	-
2018/2/21	EXP123	+	+	-	+	-
2018/2/21	EXP354	+	-	+	+	-
2018/2/21	EXP287	+	+	+	+	-
2018/2/21	EXP287	+	+	+	+	-
2018/2/22	EXP355	+	+	+	+	-
2018/2/22	EXP238	+	+	-	-	-
2018/2/23	EXP838	+	+	+	-	-
2018/2/23	EXP789	+	+	-	-	-
2018/2/27	EXP360	+	+	+	+	-
2018/2/27	EXP784	+	+	+	+	-
2018/2/28	EXP797	+	-	-	+	-
2018/2/28	EXP219	+	-	-	-	-
2018/3/1	EXP362	+	+	-	-	-
2018/3/1	EXP099	+	-	+	-	-
2018/3/1	EXP099	+	-	+	-	-
2018/3/2	EXP279	+	-	-	-	-
2018/3/2	EXP259	+	+	+	-	-
2018/3/7	EXP812	+	+	+	-	-
2018/3/8	EXP784	+	+	+	-	-
2018/3/8	EXP766	+	-	+	-	-
2018/3/8	EXP218	+	+	-	+	-
2018/3/13	EXP279	+	-	+	-	-
2018/3/13	EXP367	+	+	-	+	-
2018/3/14	EXP786	+	-	+	+	-
2018/3/15	EXP369	-	-	-	+	-
2018/3/15	EXP839	+	+	+	-	-
2018/3/15	EXP282	+	-	+	-	-
2018/3/15	EXP370	+	+	+	+	-
2018/3/15	EXP247	+	+	+	+	-
2018/3/15	EXP247	+	+	-	+	-
2018/3/15	EXP247	+	+	+	+	-
2018/3/16	EXP014	+	+	-	+	-
2018/3/16	EXP372	+	+	+	-	-
2018/3/16	EXP763	+	+	-	-	-
2018/3/19	EXP823	+	+	+	+	-
2018/3/20	EXP219	+	+	+	+	-
2018/3/21	EXP814	+	-	+	-	-
2018/3/21	EXP175	+	+	-	+	-
2018/3/21	EXP253	+	+	+	+	-
2018/3/22	EXP375	+	-	-	-	-
2018/3/22	EXP375	+	+	-	-	-
2018/3/22	EXP375	+	-	-	-	-
2018/3/23	EXP016	+	+	+	-	-
2018/3/27	EXP377	+	+	-	+	-
2018/3/27	EXP267	+	-	+	+	-
2018/3/28	EXP298	+	+	+	-	-
2018/3/28	EXP787	+	+	+	+	-
2018/3/28	EXP784	+	+	-	+	-
2018/3/28	EXP349	+	+	-	+	-
2018/4/2	EXP004	+	+	+	-	-
2018/4/5	EXP226	+	+	+	+	-
2018/4/5	EXP238	+	+	-	-	-
2018/4/6	EXP380	+	-	-	+	-
2018/4/6	EXP752	+	+	-	+	-
2018/4/10	EXP784	-	-	-	-	-
2018/4/10	EXP006	+	+	+	-	-

2018/4/10	EXP017	+	+	-	+	-
2018/4/10	EXP382	+	+	+	+	-
2018/4/10	EXP809	+	+	+	-	-
2018/4/10	EXP809	+	-	+	-	-
2018/4/11	EXP114	+	-	-	-	-
2018/4/11	EXP384	+	+	-	+	-
2018/4/11	EXP383	+	+	+	-	-
2018/4/12	EXP219	+	+	-	+	-
2018/4/13	EXP277	+	-	-	+	-
2018/4/13	EXP386	+	-	+	-	-
2018/4/13	EXP781	+	+	+	+	-
2018/4/17	EXP388	+	+	-	+	-
2018/4/17	EXP225	+	+	-	+	-
2018/4/18	EXP144	+	+	-	+	-
2018/4/18	EXP144	+	-	-	+	-
2018/4/18	EXP250	+	+	+	+	-
2018/4/19	EXP390	+	+	-	+	-
2018/4/19	EXP774	+	-	-	-	-
2018/4/19	EXP801	+	+	-	-	-
2018/4/19	EXP262	+	+	+	+	-
2018/4/20	EXP251	+	-	+	-	-
2018/4/20	EXP392	+	+	+	-	-
2018/4/24	EXP834	+	+	-	+	-
2018/4/24	EXP176	+	+	+	+	-
2018/5/2	EXP394	+	+	+	+	-
2018/5/3	EXP843	+	-	+	-	-
2018/5/3	EXP215	+	+	-	+	-
2018/5/4	EXP397	+	+	-	+	-
2018/5/8	EXP399	+	+	-	-	-
2018/5/8	EXP399	+	+	-	-	-
2018/5/8	EXP400	+	+	+	-	-
2018/5/8	EXP166	+	-	-	+	-
2018/5/8	EXP210	+	+	+	-	-
2018/5/11	EXP275	+	+	-	+	-
2018/5/15	EXP402	+	+	-	+	-
2018/5/15	EXP784	+	-	-	+	-
2018/5/17	EXP403	+	+	+	-	-
2018/5/17	EXP403	+	-	-	-	-
2018/5/18	EXP008	-	+	+	-	-
2018/5/22	EXP408	+	+	-	+	+
2018/5/22	EXP407	+	+	-	-	-
2018/5/22	EXP266	+	+	+	+	-
2018/5/22	EXP266	+	+	+	-	-
2018/5/22	EXP266	+	+	+	+	-
2018/5/23	EXP367	+	+	-	-	-
2018/5/23	EXP226	+	+	-	+	-
2018/5/24	EXP790	+	+	+	-	-
2018/5/25	EXP411	+	+	-	-	-
2018/5/28	EXP244	+	-	+	-	-
2018/5/29	EXP084	+	-	+	-	-
2018/5/29	EXP129	+	+	-	-	-
2018/5/29	EXP129	+	+	+	-	-
2018/5/29	EXP251	+	-	+	-	-
2018/5/31	EXP186	+	+	-	+	-
2018/6/1	EXP367	+	+	-	+	-
2018/6/1	EXP367	+	-	-	-	-
2018/6/1	EXP413	+	+	+	-	-
2018/6/5	EXP415	+	+	+	+	-

2018/6/5	EXP092	+	+	+	-	-
2018/6/6	EXP034	+	+	+	-	-
2018/6/6	EXP418	+	-	+	-	-
2018/6/6	EXP418	+	+	+	-	-
2018/6/6	EXP091	+	-	+	-	-
2018/6/6	EXP417	+	+	+	-	-
2018/6/6	EXP349	+	+	-	+	-
2018/6/8	EXP787	+	+	-	+	-
2018/6/8	EXP420	+	-	+	-	-
2018/6/8	EXP393	+	-	+	-	-
2018/6/8	EXP254	+	+	-	+	-
2018/6/12	EXP084	-	+	+	+	-
2018/6/12	EXP819	+	+	+	+	-
2018/6/13	EXP792	+	-	+	-	-
2018/6/13	EXP792	+	+	+	-	-
2018/6/15	EXP001	+	+	-	-	-
2018/6/15	EXP018	-	-	-	-	-
2018/6/15	EXP424	+	+	+	-	-
2018/6/15	EXP425	+	-	+	-	-
2018/6/15	EXP425	-	-	+	-	-
2018/6/19	EXP084	+	+	-	-	+
2018/6/19	EXP427	-	-	+	-	-
2018/6/19	EXP428	+	+	-	+	-
2018/6/21	EXP387	+	+	-	-	-
2018/6/21	EXP387	+	+	-	+	-
2018/6/27	EXP187	+	+	-	+	-
2018/7/4	EXP025	+	+	-	+	-
2018/7/5	EXP433	+	+	-	+	-
2018/7/10	EXP245	+	+	+	-	-
2018/7/11	EXP025	+	+	-	+	-
2018/7/13	EXP366	-	+	-	-	-
2018/7/17	EXP186	+	+	-	-	-
2018/7/17	EXP825	+	+	+	-	-
2018/7/20	EXP438	+	-	+	-	-
2018/7/20	EXP262	+	+	+	+	-
2018/7/20	EXP795	+	+	-	+	-
2018/7/25	EXP123	+	+	+	+	-
2018/7/26	EXP253	+	+	+	-	-
2018/7/31	EXP401	-	-	+	-	+
2018/8/1	EXP099	+	+	+	-	-
2018/8/1	EXP446	+	+	-	+	-
2018/8/1	EXP150	+	+	+	-	-
2018/8/2	EXP114	+	+	+	+	-
2018/8/2	EXP775	+	+	-	+	-
2018/8/9	EXP279	+	-	-	-	-
2018/8/9	EXP393	+	-	-	-	-
2018/8/10	EXP134	+	-	+	-	-
2018/8/17	EXP164	-	+	-	-	-
2018/8/23	EXP790	-	-	+	-	-
2018/8/24	EXP456	+	+	-	+	-
2018/8/24	EXP455	+	+	+	-	-
2018/8/24	EXP455	+	-	+	-	-
2018/8/24	EXP194	-	+	-	+	-
2018/8/24	EXP215	+	-	-	-	-
2018/8/28	EXP457	+	-	+	-	-
2018/8/29	EXP360	+	+	+	-	-
2018/8/30	EXP459	+	+	-	-	-
2018/8/30	EXP459	+	+	-	+	-

2018/8/30	EXP786	+	+	+	-	-
2018/8/30	EXP249	+	+	-	-	-
2018/9/4	EXP393	+	-	+	-	-
2018/9/5	EXP460	+	+	+	-	-
2018/9/5	EXP009	+	-	+	-	-
2018/9/5	EXP187	+	+	-	+	-
2018/9/6	EXP463	+	-	+	-	-
2018/9/6	EXP080	+	+	+	+	-
2018/9/6	EXP129	+	+	-	-	-
2018/9/7	EXP465	+	-	+	-	-
2018/9/7	EXP310	+	-	-	-	-
2018/9/11	EXP326	+	+	+	-	-
2018/9/11	EXP215	-	-	-	-	-
2018/9/11	EXP266	+	+	+	-	-
2018/9/12	EXP467	+	-	+	-	-
2018/9/12	EXP194	-	+	+	+	-
2018/9/13	EXP084	+	-	-	-	+
2018/9/14	EXP250	-	+	-	-	-
2018/9/18	EXP470	-	-	-	-	-
2018/9/19	EXP045	-	+	-	-	-
2018/9/19	EXP045	+	+	+	-	-
2018/9/19	EXP060	+	+	+	-	-
2018/9/19	EXP411	+	-	-	-	-
2018/9/19	EXP367	+	+	-	-	-
2018/9/24	EXP245	+	-	+	-	+
2018/9/26	EXP792	+	+	+	+	-
2018/9/27	EXP453	+	+	-	+	-
2018/10/2	EXP215	+	-	-	-	-
2018/10/4	EXP473	+	+	+	-	-
2018/10/4	EXP473	+	+	+	-	-
2018/10/5	EXP267	+	+	+	-	-
2018/10/5	EXP267	+	-	-	-	-
2018/10/9	EXP044	+	+	+	-	-
2018/10/9	EXP044	+	+	-	-	-
2018/10/9	EXP064	-	+	-	-	-
2018/10/10	EXP131	+	-	-	-	-
2018/10/10	EXP348	+	+	+	+	-
2018/10/10	EXP348	+	+	-	+	-
2018/10/10	EXP348	+	+	-	+	-
2018/10/15	EXP778	+	+	-	-	-
2018/10/15	EXP778	+	-	-	-	-
2018/10/17	EXP360	+	+	+	+	-
2018/10/17	EXP778	+	+	-	-	-
2018/10/17	EXP482	+	-	-	-	-
2018/10/17	EXP484	+	+	+	-	-
2018/10/18	EXP058	+	+	-	+	-
2018/10/19	EXP045	-	-	-	-	-
2018/10/19	EXP045	+	-	-	-	-
2018/10/19	EXP045	-	-	-	-	-
2018/10/19	EXP045	-	-	-	-	-
2018/10/19	EXP045	-	-	-	-	-
2018/10/19	EXP172	+	+	-	+	-
2018/10/23	EXP019	+	+	-	-	-
2018/10/23	EXP819	+	+	+	-	-
2018/10/23	EXP241	+	+	-	-	-
2018/10/25	EXP810	+	-	-	-	-
2018/10/25	EXP810	+	+	-	-	-
2018/10/25	EXP305	-	-	+	-	-

2018/10/25	EXP488	+	-	-	-	-
2018/10/26	EXP490	+	+	+	-	-
2018/10/26	EXP490	+	+	+	+	-
2018/10/31	EXP420	+	-	-	-	-
2018/11/5	EXP187	+	+	+	+	-
2018/11/6	EXP254	+	+	-	+	-
2018/11/6	EXP267	+	+	-	-	-
2018/11/7	EXP367	+	+	-	+	-
2018/11/8	EXP494	-	-	+	+	+
2018/11/9	EXP018	-	-	-	-	-
2018/11/12	EXP493	+	+	+	-	-
2018/11/13	EXP838	+	+	-	-	-
2018/11/13	EXP839	+	-	-	-	-
2018/11/14	EXP497	-	+	+	+	-
2018/11/14	EXP497	+	+	-	-	-
2018/11/14	EXP497	-	+	-	+	-
2018/11/14	EXP497	+	+	-	-	-
2018/11/14	EXP497	+	+	-	+	-
2018/11/14	EXP497	-	-	-	-	-
2018/11/15	EXP131	+	+	-	-	-
2018/11/20	EXP019	-	+	-	-	-
2018/11/20	EXP500	+	+	-	-	-
2018/11/20	EXP813	+	+	-	+	-
2018/11/21	EXP265	+	+	-	-	-
2018/11/22	EXP041	+	+	-	-	-
2018/11/22	EXP497	-	+	-	-	-
2018/11/22	EXP497	-	+	+	-	-
2018/11/22	EXP497	-	+	+	-	-
2018/11/22	EXP497	-	+	+	+	-
2018/11/22	EXP497	-	+	-	-	-
2018/11/22	EXP497	-	+	+	-	-
2018/11/22	EXP367	+	+	-	+	-
2018/11/22	EXP501	+	+	-	+	-
2018/11/22	EXP501	+	+	+	-	+
2018/11/23	EXP420	+	+	-	+	-
2018/11/23	EXP502	+	+	+	+	-
2018/11/27	EXP837	+	+	-	-	-
2018/11/28	EXP464	-	-	+	-	-
2018/11/28	EXP075	+	-	-	+	-
2018/11/28	EXP505	+	+	+	-	-
2018/12/4	EXP508	+	-	+	-	-
2018/12/4	EXP255	+	+	-	+	-
2018/12/4	EXP780	+	-	+	-	-
2018/12/5	EXP041	+	-	+	-	-
2018/12/7	EXP497	-	+	-	-	-
2018/12/7	EXP497	+	-	-	-	-
2018/12/7	EXP497	+	+	-	-	-
2018/12/7	EXP497	+	-	-	-	-
2018/12/7	EXP497	+	+	-	-	-
2018/12/7	EXP497	-	+	-	-	-
2018/12/7	EXP153	+	+	-	-	-
2018/12/7	EXP253	+	+	+	-	-
2018/12/7	EXP262	+	+	+	-	-
2018/12/7	EXP795	+	-	-	-	-
2018/12/7	EXP825	+	+	+	-	-
2018/12/13	EXP523	+	+	-	+	-
2018/12/13	EXP764	+	+	-	+	-
2018/12/13	EXP792	+	+	+	+	-

2018/12/14	EXP063	+	+	+	-	-
2018/12/17	EXP265	+	+	+	-	-
2018/12/18	EXP838	+	+	-	-	-
2018/12/18	EXP235	+	+	-	+	-
2018/12/19	EXP031	+	+	+	-	-
2018/12/20	EXP151	+	-	+	-	-
2018/12/21	EXP395	+	-	-	-	-
2018/12/21	EXP437	+	+	+	-	-
2018/12/27	EXP263	+	-	-	-	-
2019/1/3	EXP215	+	-	-	-	+
2019/1/4	EXP508	-	-	-	-	-
2019/1/9	EXP039	-	-	-	-	-
2019/1/9	EXP534	+	+	-	+	-
2019/1/9	EXP533	+	+	-	+	-
2019/1/9	EXP241	+	+	-	+	-
2019/1/9	EXP241	+	+	-	+	-
2019/1/9	EXP245	+	-	+	-	-
2019/1/10	EXP472	+	-	-	+	-
2019/1/10	EXP472	+	+	-	-	-
2019/1/10	EXP123	+	+	-	+	-
2019/1/10	EXP224	-	-	-	-	-
2019/1/11	EXP033	-	-	+	-	-
2019/1/11	EXP033	-	-	+	-	-
2019/1/11	EXP539	+	+	-	+	-
2019/1/11	EXP790	+	+	-	-	-
2019/1/11	EXP250	+	+	-	+	-
2019/1/14	EXP268	+	+	-	+	-
2019/1/15	EXP029	+	-	-	-	-
2019/1/15	EXP149	+	+	+	-	-
2019/1/15	EXP349	+	+	+	-	-
2019/1/15	EXP239	+	-	-	-	-
2019/1/15	EXP825	+	+	+	+	-
2019/1/16	EXP546	+	+	-	-	-
2019/1/16	EXP416	+	+	-	+	-
2019/1/16	EXP249	+	+	-	+	-
2019/1/17	EXP205	+	+	+	-	-
2019/1/17	EXP215	+	+	-	-	+
2019/1/18	EXP222	+	+	+	+	-
2019/1/18	EXP792	+	+	+	-	-
2019/1/22	EXP067	+	+	-	+	-
2019/1/22	EXP124	+	+	+	-	-
2019/1/22	EXP549	+	-	+	-	-
2019/1/22	EXP549	+	-	+	-	-
2019/1/22	EXP219	+	+	+	-	-
2019/1/23	EXP551	+	+	-	-	-
2019/1/23	EXP479	+	+	-	-	-
2019/1/23	EXP310	+	-	-	-	-
2019/1/24	EXP497	+	-	+	-	-
2019/1/24	EXP497	-	-	+	-	-
2019/1/24	EXP497	+	-	+	-	-
2019/1/24	EXP497	-	-	+	-	-
2019/1/24	EXP497	-	+	+	-	-
2019/1/24	EXP497	+	+	+	-	-
2019/1/25	EXP063	+	+	-	-	-
2019/1/28	EXP239	+	+	-	-	-
2019/1/29	EXP091	+	+	-	+	-
2019/1/29	EXP193	+	+	-	-	-
2019/1/31	EXP166	-	+	-	+	-

2019/1/31	EXP832	+	+	+	+	-
2019/1/31	EXP558	+	+	+	-	-
2019/1/31	EXP265	+	+	-	+	-
2019/2/1	EXP069	+	+	+	-	-
2019/2/1	EXP267	+	-	-	-	-
2019/2/5	EXP564	+	-	-	-	-
2019/2/5	EXP313	+	+	+	-	-
2019/2/6	EXP131	+	+	-	-	-
2019/2/6	EXP342	+	-	+	+	-
2019/2/6	EXP342	-	-	+	-	-
2019/2/7	EXP367	+	+	-	-	-
2019/2/7	EXP157	+	-	+	-	-
2019/2/7	EXP273	+	-	-	+	+
2019/2/7	EXP273	+	-	-	-	+
2019/2/8	EXP048	+	-	+	-	-
2019/2/8	EXP810	+	-	-	-	-
2019/2/8	EXP753	+	+	-	+	-
2019/2/11	EXP096	+	+	+	-	-
2019/2/12	EXP306	+	+	-	+	-
2019/2/12	EXP100	+	+	+	-	-
2019/2/12	EXP409	+	+	-	+	-
2019/2/13	EXP778	+	+	-	-	-
2019/2/13	EXP805	+	+	-	+	-
2019/2/13	EXP232	+	-	+	-	-
2019/2/13	EXP255	+	+	-	-	-
2019/2/14	EXP815	-	-	+	-	-
2019/2/15	EXP573	+	+	-	+	-
2019/2/15	EXP309	+	+	-	+	-
2019/2/18	EXP223	+	+	+	-	-
2019/2/18	EXP313	+	+	+	-	-
2019/2/19	EXP038	+	+	+	-	-
2019/2/19	EXP574	+	+	+	+	-
2019/2/19	EXP210	+	+	+	-	-
2019/2/20	EXP279	+	+	+	-	-
2019/2/20	EXP776	+	+	-	+	-
2019/2/21	EXP086	+	+	-	-	-
2019/2/21	EXP827	+	+	+	-	-
2019/2/22	EXP166	-	+	-	-	-
2019/2/22	EXP223	+	+	+	-	-
2019/2/22	EXP223	+	+	+	-	-
2019/2/22	EXP223	+	+	+	-	-
2019/2/22	EXP548	+	+	-	+	-
2019/2/22	EXP580	+	+	+	-	-
2019/2/26	EXP549	+	-	-	-	-
2019/2/26	EXP819	+	+	-	-	-
2019/2/26	EXP398	+	+	-	-	-
2019/2/26	EXP240	+	+	+	+	-
2019/2/26	EXP240	-	-	+	-	-
2019/2/26	EXP760	+	+	+	-	-
2019/2/27	EXP052	+	-	+	+	-
2019/2/27	EXP064	+	+	-	+	-
2019/2/27	EXP762	+	+	-	+	-
2019/2/27	EXP582	+	+	-	+	-
2019/2/28	EXP065	+	+	-	-	-
2019/2/28	EXP583	+	+	+	-	-
2019/2/28	EXP206	-	-	+	-	-
2019/3/1	EXP585	+	-	-	-	-
2019/3/1	EXP796	+	+	+	+	-

2019/3/4	EXP265	+	-	+	+	-
2019/3/4	EXP265	+	+	+	-	-
2019/3/4	EXP265	+	-	-	-	-
2019/3/4	EXP265	+	+	+	+	-
2019/3/5	EXP176	+	+	+	+	-
2019/3/5	EXP479	+	+	+	-	-
2019/3/6	EXP497	+	-	-	+	-
2019/3/6	EXP497	+	-	-	+	-
2019/3/6	EXP497	+	+	-	+	-
2019/3/6	EXP497	-	-	-	+	-
2019/3/6	EXP497	+	-	-	-	-
2019/3/6	EXP497	+	+	-	+	-
2019/3/6	EXP497	+	-	-	+	-
2019/3/6	EXP497	+	-	+	+	-
2019/3/6	EXP497	+	-	+	+	-
2019/3/6	EXP497	+	+	-	-	-
2019/3/6	EXP497	-	-	-	+	-
2019/3/6	EXP497	+	+	-	+	-
2019/3/7	EXP250	+	+	-	-	-
2019/3/8	EXP027	+	+	-	-	-
2019/3/8	EXP790	-	-	+	-	-
2019/3/8	EXP127	+	+	+	-	-
2019/3/8	EXP149	+	+	-	+	-
2019/3/8	EXP580	+	+	+	-	-
2019/3/8	EXP580	+	+	+	-	-
2019/3/11	EXP129	-	+	-	-	-
2019/3/12	EXP128	+	+	-	+	-
2019/3/12	EXP266	+	-	+	-	-
2019/3/12	EXP589	+	+	+	-	-
2019/3/12	EXP589	+	+	+	-	-
2019/3/12	EXP589	+	+	+	-	-
2019/3/12	EXP589	+	-	-	-	-
2019/3/13	EXP762	+	+	+	+	-
2019/3/13	EXP149	+	+	-	+	-
2019/3/13	EXP824	+	+	-	+	-
2019/3/13	EXP824	+	-	-	+	-
2019/3/13	EXP824	+	+	-	+	-
2019/3/13	EXP824	+	+	-	+	-
2019/3/13	EXP824	+	+	-	-	-
2019/3/14	EXP084	+	+	-	-	-
2019/3/14	EXP140	+	+	-	+	-
2019/3/14	EXP409	+	-	-	+	-
2019/3/14	EXP409	+	-	-	-	-
2019/3/14	EXP594	+	+	-	+	-
2019/3/15	EXP020	+	-	+	-	-
2019/3/15	EXP065	+	+	+	-	-
2019/3/15	EXP597	+	+	+	-	+
2019/3/15	EXP596	+	-	+	-	+
2019/3/15	EXP206	+	+	+	-	-
2019/3/19	EXP843	+	+	+	-	-
2019/3/19	EXP102	-	-	+	-	-
2019/3/19	EXP769	+	-	-	-	-
2019/3/19	EXP315	+	-	-	-	-
2019/3/20	EXP029	+	-	-	-	-
2019/3/20	EXP031	-	-	+	-	-
2019/3/20	EXP412	+	+	+	-	-
2019/3/20	EXP600	-	-	+	+	-
2019/3/21	EXP183	+	+	-	-	-

2019/3/21	EXP257	+	+	+	-	-
2019/3/21	EXP257	-	-	+	-	-
2019/3/22	EXP091	+	+	-	+	-
2019/3/26	EXP549	+	-	+	-	-
2019/3/26	EXP479	+	+	+	-	-
2019/3/26	EXP245	+	+	+	-	-
2019/3/26	EXP254	+	+	+	-	-
2019/3/27	EXP024	+	+	-	+	-
2019/3/27	EXP058	+	-	+	-	-
2019/3/27	EXP139	+	+	-	-	-
2019/3/27	EXP605	+	+	-	+	-
2019/3/27	EXP428	+	+	-	+	-
2019/3/28	EXP034	+	+	-	+	-
2019/3/28	EXP360	+	-	-	-	-
2019/3/28	EXP787	+	+	+	+	-
2019/3/28	EXP079	+	+	-	-	-
2019/3/28	EXP079	+	-	-	-	-
2019/3/28	EXP778	+	-	-	-	-
2019/3/28	EXP166	+	+	-	-	-
2019/3/28	EXP171	+	+	+	-	-
2019/3/28	EXP779	+	+	-	+	-
2019/3/29	EXP606	-	-	+	+	-
2019/3/29	EXP084	+	+	+	-	-
2019/3/29	EXP160	+	+	-	-	-
2019/4/2	EXP575	+	+	-	-	-
2019/4/2	EXP167	+	-	+	-	-
2019/4/2	EXP266	-	-	-	-	+
2019/4/3	EXP497	+	-	-	-	-
2019/4/3	EXP497	-	-	-	-	-
2019/4/3	EXP497	+	-	-	-	-
2019/4/3	EXP497	+	-	-	+	-
2019/4/3	EXP497	+	-	-	-	-
2019/4/3	EXP497	+	-	-	-	-
2019/4/3	EXP497	-	-	-	-	-
2019/4/3	EXP497	-	-	-	-	-
2019/4/3	EXP497	+	+	-	+	-
2019/4/3	EXP497	+	-	-	-	-
2019/4/3	EXP605	+	+	-	+	-
2019/4/3	EXP287	+	+	-	+	-
2019/4/4	EXP169	+	+	-	+	-
2019/4/4	EXP808	+	-	+	-	-
2019/4/5	EXP035	-	-	+	-	-
2019/4/5	EXP171	+	+	-	+	-
2019/4/5	EXP269	+	+	+	-	-
2019/4/9	EXP750	+	-	-	+	-
2019/4/9	EXP215	+	-	-	-	+
2019/4/10	EXP129	+	+	+	+	-
2019/4/10	EXP129	+	+	+	+	-
2019/4/11	EXP612	+	+	-	+	-
2019/4/11	EXP793	+	-	-	+	-
2019/4/11	EXP497	+	-	-	-	-
2019/4/11	EXP497	+	-	-	-	-
2019/4/11	EXP497	-	-	-	-	-
2019/4/11	EXP208	+	+	+	-	-
2019/4/11	EXP235	+	+	-	+	-
2019/4/11	EXP287	+	-	+	-	-
2019/4/12	EXP563	+	+	-	-	-
2019/4/12	EXP776	+	-	-	+	-

2019/4/12	EXP135	+	-	+	-	-
2019/4/12	EXP614	+	+	+	-	-
2019/4/16	EXP497	-	-	-	-	-
2019/4/16	EXP497	-	+	-	-	-
2019/4/16	EXP497	+	-	-	-	-
2019/4/16	EXP497	+	-	-	-	-
2019/4/16	EXP220	+	+	-	-	-
2019/4/17	EXP008	-	-	-	-	-
2019/4/24	EXP628	-	-	-	-	-
2019/4/26	EXP112	+	+	+	-	-
2019/4/26	EXP236	+	+	-	+	-
2019/4/30	EXP132	+	+	-	-	-
2019/5/2	EXP622	+	+	-	+	-
2019/5/2	EXP621	+	+	+	-	-
2019/5/2	EXP227	+	+	+	+	-
2019/5/3	EXP623	+	-	-	+	-
2019/5/7	EXP171	-	+	+	-	-
2019/5/8	EXP025	+	-	+	-	-
2019/5/8	EXP625	+	-	-	-	-
2019/5/9	EXP224	+	+	-	-	-
2019/5/10	EXP040	+	+	+	-	-
2019/5/10	EXP588	+	+	-	-	-
2019/5/13	EXP254	+	+	+	-	-
2019/5/15	EXP477	+	+	-	+	-
2019/5/15	EXP786	+	-	-	-	-
2019/5/16	EXP802	+	+	+	+	-
2019/5/17	EXP239	+	+	+	+	-
2019/5/17	EXP239	+	+	-	-	-
2019/5/21	EXP632	+	+	-	+	-
2019/5/21	EXP225	+	+	-	+	-
2019/5/22	EXP633	+	-	-	-	+
2019/5/22	EXP634	+	-	+	-	-
2019/5/22	EXP220	+	+	-	+	-
2019/5/23	EXP598	+	-	-	+	-
2019/5/24	EXP636	+	-	-	-	-
2019/5/28	EXP637	+	+	-	-	-
2019/5/28	EXP157	+	+	-	-	+
2019/5/28	EXP461	+	+	+	+	-
2019/5/29	EXP221	+	+	-	+	-
2019/5/30	EXP291	+	+	+	+	-
2019/5/30	EXP600	+	+	+	-	-
2019/5/31	EXP056	+	+	-	+	-
2019/5/31	EXP166	+	+	-	-	-
2019/6/4	EXP185	+	+	-	-	-
2019/6/5	EXP642	-	-	-	-	-
2019/6/5	EXP643	-	-	-	-	-
2019/6/5	EXP177	+	+	+	-	-
2019/6/5	EXP211	+	+	-	+	-
2019/6/7	EXP646	+	+	-	+	-
2019/6/10	EXP520	+	-	+	-	-
2019/6/10	EXP821	-	-	-	-	-
2019/6/11	EXP366	+	+	+	+	-
2019/6/12	EXP149	+	+	-	-	-
2019/6/12	EXP248	+	-	-	+	-
2019/6/12	EXP248	+	-	-	-	-
2019/6/12	EXP248	+	-	-	-	-
2019/6/14	EXP065	+	+	+	-	-
2019/6/14	EXP651	-	-	-	-	-

2019/6/14	EXP286	+	+	-	-	-
2019/6/18	EXP091	+	+	-	+	-
2019/6/18	EXP653	+	-	+	-	-
2019/6/18	EXP149	+	+	-	-	-
2019/6/18	EXP253	+	+	+	-	-
2019/6/19	EXP654	+	+	-	-	-
2019/6/19	EXP770	+	-	+	-	-
2019/6/20	EXP769	+	+	-	-	+
2019/6/21	EXP655	+	+	+	-	-
2019/6/21	EXP844	+	+	-	-	-
2019/6/21	EXP267	+	+	-	-	-
2019/6/21	EXP267	+	-	-	-	-
2019/6/25	EXP842	+	+	-	+	-
2019/6/26	EXP657	+	+	+	-	-
2019/6/26	EXP153	+	-	+	+	-
2019/6/27	EXP658	+	+	-	-	-
2019/6/28	EXP659	+	-	+	+	+
2019/7/2	EXP174	-	-	+	-	-
2019/7/3	EXP055	-	-	+	-	-
2019/7/3	EXP325	+	-	-	-	-
2019/7/3	EXP325	+	-	-	-	-
2019/7/5	EXP751	+	-	-	-	-
2019/7/5	EXP082	+	+	+	-	-
2019/7/5	EXP084	+	+	-	+	-
2019/7/8	EXP520	+	+	+	+	-
2019/7/9	EXP666	-	-	+	+	+
2019/7/9	EXP665	-	-	+	-	-
2019/7/9	EXP248	+	-	-	-	-
2019/7/11	EXP300	+	+	+	-	-
2019/7/12	EXP147	-	-	+	-	-
2019/7/16	EXP457	+	+	+	-	-
2019/7/17	EXP322	+	+	+	-	-
2019/7/17	EXP322	+	+	+	-	-
2019/7/17	EXP322	-	-	-	-	-
2019/7/17	EXP322	+	-	-	-	-
2019/7/17	EXP322	+	+	+	-	-
2019/7/17	EXP322	+	+	+	-	-
2019/7/17	EXP672	+	-	-	-	-
2019/7/17	EXP672	+	-	-	-	-
2019/7/18	EXP084	+	+	+	-	-
2019/7/18	EXP239	+	+	-	+	-
2019/7/18	EXP239	+	+	-	+	-
2019/7/18	EXP250	+	+	-	-	+
2019/7/19	EXP784	+	-	-	-	-
2019/7/19	EXP773	+	+	+	-	-
2019/7/22	EXP119	+	+	+	+	-
2019/7/24	EXP022	+	-	+	-	-
2019/7/24	EXP072	-	-	+	-	-
2019/7/26	EXP131	+	+	-	-	-
2019/7/26	EXP131	+	+	-	-	-
2019/7/26	EXP131	+	-	-	-	-
2019/7/26	EXP131	+	-	-	-	-
2019/7/30	EXP063	+	-	-	-	-
2019/7/30	EXP188	+	-	+	-	-
2019/7/30	EXP188	+	-	+	-	-
2019/7/30	EXP188	+	+	+	-	-
2019/7/30	EXP265	+	+	-	-	-
2019/7/31	EXP360	+	-	+	-	-

2019/7/31	EXP676	+	+	-	+	-
2019/7/31	EXP276	+	+	-	-	-
2019/7/31	EXP675	+	-	+	-	+
2019/7/31	EXP499	+	-	+	-	-
2019/7/31	EXP821	+	+	+	-	-
2019/8/1	EXP678	+	+	-	+	-
2019/8/1	EXP145	+	+	-	-	-
2019/8/1	EXP273	+	+	+	-	-
2019/8/2	EXP643	+	+	-	-	-
2019/8/2	EXP679	+	-	-	-	-
2019/8/7	EXP084	+	+	+	-	-
2019/8/7	EXP172	+	-	+	-	-
2019/8/7	EXP172	+	+	+	-	-
2019/8/8	EXP683	+	-	-	+	-
2019/8/8	EXP683	+	-	-	+	-
2019/8/14	EXP753	+	+	+	-	-
2019/8/14	EXP753	+	+	-	-	-
2019/8/14	EXP165	+	+	-	+	-
2019/8/16	EXP756	+	+	-	-	-
2019/8/19	EXP266	+	-	-	-	-
2019/8/21	EXP531	+	+	-	+	-
2019/8/23	EXP683	+	+	-	+	-
2019/8/23	EXP688	+	+	+	-	-
2019/8/23	EXP172	+	+	-	+	-
2019/8/28	EXP084	+	+	-	-	-
2019/8/29	EXP086	+	-	+	-	-
2019/8/30	EXP690	+	+	+	-	-
2019/9/3	EXP093	+	+	+	-	-
2019/9/3	EXP261	+	+	-	-	+
2019/9/4	EXP214	+	+	-	-	-
2019/9/5	EXP091	+	+	-	+	-
2019/9/6	EXP641	+	-	-	-	-
2019/9/9	EXP193	+	-	-	-	-
2019/9/10	EXP085	+	+	+	-	-
2019/9/11	EXP057	+	-	-	-	-
2019/9/11	EXP057	+	-	+	-	-
2019/9/11	EXP277	+	+	+	-	-
2019/9/11	EXP145	+	+	-	-	-
2019/9/13	EXP101	+	+	-	+	-
2019/9/17	EXP129	+	+	-	+	-
2019/9/17	EXP129	+	-	-	-	-
2019/9/18	EXP235	+	+	-	-	-
2019/9/18	EXP235	+	+	-	-	-
2019/9/18	EXP239	+	+	-	-	-
2019/9/18	EXP239	+	+	-	-	-
2019/9/19	EXP819	+	+	+	+	-
2019/9/20	EXP698	+	+	-	-	-
2019/9/20	EXP420	+	-	+	-	-
2019/9/20	EXP420	-	-	+	+	+
2019/9/20	EXP128	+	-	+	-	-
2019/9/20	EXP188	+	+	+	-	-
2019/9/20	EXP188	-	-	+	-	+
2019/9/20	EXP247	+	+	+	-	-
2019/9/20	EXP264	+	+	+	-	-
2019/9/23	EXP792	+	+	-	-	-
2019/9/24	EXP179	-	-	+	-	-
2019/9/25	EXP136	+	-	-	+	-
2019/9/25	EXP701	+	-	+	-	-

2019/9/26	EXP276	+	+	-	-	-
2019/9/26	EXP264	+	+	+	-	-
2019/9/26	EXP752	+	+	-	-	-
2019/9/26	EXP752	+	+	+	-	-
2019/9/27	EXP187	+	+	-	-	-
2019/9/27	EXP263	+	+	+	-	-
2019/10/1	EXP706	+	-	-	-	-
2019/10/2	EXP707	+	-	-	-	-
2019/10/2	EXP707	+	-	-	-	-
2019/10/4	EXP279	-	-	-	-	-
2019/10/4	EXP657	+	+	+	-	-
2019/10/4	EXP708	+	+	-	+	-
2019/10/8	EXP477	+	+	+	+	-
2019/10/8	EXP162	+	+	-	-	-
2019/10/9	EXP709	+	-	+	-	-
2019/10/9	EXP308	+	-	+	+	+
2019/10/9	EXP308	-	-	+	-	-
2019/10/10	EXP149	+	+	-	+	-
2019/10/10	EXP160	+	+	-	+	-
2019/10/11	EXP217	+	+	-	-	-
2019/10/11	EXP265	+	+	-	-	-
2019/10/11	EXP266	+	-	+	-	+
2019/10/15	EXP078	+	+	-	-	-
2019/10/15	EXP713	+	-	-	+	-
2019/10/16	EXP790	+	-	-	-	-
2019/10/16	EXP123	+	+	-	+	-
2019/10/16	EXP291	+	+	+	-	-
2019/10/16	EXP173	+	-	-	-	-
2019/10/16	EXP188	+	+	+	-	-
2019/10/22	EXP716	+	-	-	-	-
2019/10/23	EXP107	+	-	+	-	-
2019/10/23	EXP717	+	+	-	-	-
2019/10/23	EXP717	-	-	-	-	-
2019/10/24	EXP720	+	+	+	+	-
2019/10/24	EXP397	+	-	-	+	-
2019/10/24	EXP308	+	+	-	+	-
2019/10/25	EXP263	+	+	+	-	-
2019/10/25	EXP800	+	+	+	-	+
2019/10/30	EXP098	+	+	-	-	-
2019/10/30	EXP098	+	-	-	-	-
2019/10/31	EXP722	+	+	-	+	-
2019/10/31	EXP360	+	-	-	-	-
2019/10/31	EXP533	+	+	+	-	-
2019/10/31	EXP688	+	-	-	-	-
2019/10/31	EXP178	+	+	-	-	-
2019/11/6	EXP186	+	-	-	-	-
2019/11/6	EXP186	+	-	+	-	-
2019/11/7	EXP726	+	+	-	-	-
2019/11/8	EXP013	+	+	-	-	-
2019/11/8	EXP758	+	+	+	-	-
2019/11/8	EXP758	+	+	+	-	-
2019/11/12	EXP800	+	+	+	-	-
2019/11/13	EXP753	+	+	+	-	-
2019/11/15	EXP803	+	+	+	-	-
2019/11/15	EXP068	+	-	-	+	-
2019/11/18	EXP221	+	+	-	+	-
2019/11/18	EXP221	+	+	-	+	-
2019/11/19	EXP767	+	-	+	+	-

2019/11/19	EXP279	+	+	+	-	-
2019/11/19	EXP193	+	-	+	-	-
2019/11/19	EXP266	+	-	+	-	+
2019/11/19	EXP266	+	-	+	-	-
2019/11/20	EXP142	+	-	+	-	-
2019/11/21	EXP129	+	+	-	-	-
2019/11/21	EXP349	+	+	-	-	-
2019/11/21	EXP246	+	+	+	+	-
2019/11/21	EXP246	+	+	+	-	+
2019/11/21	EXP263	+	-	+	+	-
2019/11/22	EXP668	+	-	-	-	-
2019/11/25	EXP111	-	-	+	-	+
2019/11/26	EXP062	+	+	-	-	-
2019/11/26	EXP836	+	+	-	-	-
2019/11/27	EXP128	+	+	-	+	-
2019/11/27	EXP005	+	-	-	-	-
2019/11/27	EXP181	+	+	-	+	-
2019/12/2	EXP732	+	+	+	-	-
2019/12/2	EXP187	+	+	-	+	-
2019/12/3	EXP190	+	+	-	+	-
2019/12/4	EXP065	+	+	+	-	-
2019/12/5	EXP053	+	-	+	-	-
2019/12/5	EXP249	+	+	-	+	-
2019/12/5	EXP825	+	+	-	-	-
2019/12/10	EXP136	+	+	-	+	-
2019/12/11	EXP049	-	-	+	+	-
2019/12/11	EXP174	+	-	-	-	+
2019/12/11	EXP226	+	-	+	-	-
2019/12/11	EXP263	+	+	+	-	-
2019/12/11	EXP263	+	+	+	-	-
2019/12/12	EXP011	+	+	-	+	-
2019/12/12	EXP106	+	-	-	-	-
2019/12/12	EXP737	+	+	-	-	-
2019/12/13	EXP070	+	-	-	-	-
2019/12/13	EXP070	+	-	+	-	-
2019/12/13	EXP153	+	+	-	+	-
2019/12/13	EXP153	+	+	-	+	-
2019/12/17	EXP079	+	-	+	-	-
2019/12/17	EXP111	+	+	-	+	-
2019/12/17	EXP111	+	-	+	-	+
2019/12/18	EXP738	+	+	-	+	-
2019/12/18	EXP222	+	+	+	-	-
2019/12/18	EXP266	-	-	-	-	+
2019/12/19	EXP739	+	-	-	-	-
2019/12/20	EXP843	+	+	-	+	-
2019/12/20	EXP741	+	-	-	-	-
2019/12/20	EXP187	+	+	-	-	+
2019/12/20	EXP223	+	+	-	+	-
2019/12/20	EXP223	+	+	-	+	-
2019/12/20	EXP313	+	-	+	-	-
2019/12/20	EXP313	+	+	+	-	-
2019/12/20	EXP315	+	+	+	-	-
2019/12/20	EXP315	+	+	-	-	-
2019/12/27	EXP187	+	+	-	+	+
2019/12/30	EXP187	+	+	-	+	+
2019/12/31	EXP688	+	+	+	-	-
2019/12/31	EXP743	+	+	+	-	-
2019/12/31	EXP244	+	-	-	+	-
