# Supplementary material for: Occurrence of Rotavirus A Genotypes and Other Enteric Pathogens in Diarrheic Suckling Piglets from Spanish Swine Farms
Source: Animals (Basel). 2022 Jan 20;12(3):251. doi: 10.3390/ani12030251 (PMC8833434; doi:10.3390/ani12030251)
Supplement: Supplementary file 1 [file animals-12-00251-s001.zip › Supplementary Tables S2-S13.pdf]

Table S2. Nucleotide similarity (%) among RVA strains of the G4 genotype found in the current study and the prototype strain contained in the vaccine available in Spain (RVA/Pig-tc/USA/LS00007_Gottfried/1975/G4P6) (lower semi-matrix). Similarity among the amino acids coded by the same sequences is indicated in bold in the upper semi-matrix. Identity of RVA strains according to the nomenclature proposed by the RCWG is indicated in Table S10.

Nucleotides	Amino acids																									
Strain ID	**1**	**2**	**3**	**4**	**5**	**6**	**7**	**8**	**9**	**10**	**11**	**12**	**13**	**14**	**15**	**16**	**17**	**18**	**19**	**20**	**21**	**22**	**23**	**24**	**25**	**13**
1		99.7	98.5	98.5	98.8	97.5	98.2	98.5	98.5	97.2	97.9	97.6	98.5	97.2	96.9	96.3	95.4	95.4	94.2	94.4	94.2	95.4	88.0	89.3	89.4	92.9
2	99.9		98.8	98.8	99.1	97.8	98.5	98.8	98.9	97.6	98.2	97.9	98.8	97.6	97.2	96.6	95.7	95.7	94.5	94.4	94.5	95.8	88.3	89.6	89.2	93.3
3	99.3	93.4		97.8	97.8	96.9	97.5	97.5	98.1	96.6	97.2	96.9	97.5	96.3	96.0	96.0	95.1	95.1	93.6	93.7	93.9	95.4	87.4	88.7	89.1	92.6
4	98.5	98.6	97.9		99.7	98.5	99.1	99.4	98.5	98.2	98.8	98.2	99.4	97.2	96.9	97.2	95.7	95.7	95.4	95.3	95.4	96.8	89.3	90.5	90.5	94.2
5	98.3	98.4	97.8	98.9		98.2	98.8	99.7	98.9	97.9	98.5	98.2	99.7	97.6	97.2	97.2	95.7	95.7	95.4	95.3	95.4	96.8	89.0	90.2	90.1	94.2
6	98.1	98.2	97.5	98.6	98.2		98.2	97.8	98.1	99.7	98.5	99.7	97.9	96.3	96.0	95.7	94.8	94.8	94.2	94.0	93.9	95.8	89.0	90.2	90.1	93.3
7	98.1	98.2	97.5	98.6	98.2	98.2		98.5	98.9	97.9	98.5	97.9	98.5	97.6	97.2	96.9	96.0	96.0	95.1	95.0	95.1	95.8	88.7	89.9	89.8	93.9
8	97.9	98.1	97.4	98.7	99.7	97.9	97.9		98.5	97.6	98.2	97.9	99.4	97.2	96.9	96.9	95.4	95.4	95.1	95.0	95.1	96.5	88.7	89.9	89.8	93.9
9	97.9	97.9	97.3	98.1	98.2	97.9	97.8	97.9		97.6	98.1	97.8	98.2	96.9	96.6	96.3	95.4	95.4	94.5	94.4	94.5	95.4	89.0	90.2	90.1	92.9
10	97.9	97.9	97.3	98.4	97.9	99.8	97.9	97.7	97.8		98.2	99.4	96.0	96.0	95.7	95.4	94.5	94.5	93.9	93.7	93.6	95.4	88.7	89.9	89.8	92.9
11	97.7	97.8	97.1	98.2	97.8	98.6ç	97.8	97.4	97.4	98.4		98.2	98.2	97.2	96.9	96.0	95.4	95.1	94.8	94.7	94.8	95.4	88.3	89.6	89.4	93.3
12	97.7	97.8	97.3	98.2	97.9	99.6	97.8	97.7	97.6	99.4	98.2		97.9	96.3	96.0	95.7	94.8	94.8	94.5	94.0	93.9	95.8	88.7	89.9	89.8	93.3
13	97.8	97.9	97.3	98.6	98.9	97.9	97.7	98.7	97.5	97.7	97.7	97.7		97.2	96.9	96.9	95.4	95.4	95.1	95.0	95.1	96.5	88.6	89.9	89.8	93.9
14	95.3	95.4	95.0	95.8	95.7	95.6	95.8	95.4	95.2	95.4	95.4	95.2	95.6		99.7	96.6	95.7	95.7	95.4	95.6	95.4	95.4	90.0	89.9	89.8	93.6
15	95.2	95.3	94.9	95.7	95.6	95.5	95.7	95.3	95.1	95.3	95.3	92.2	95.5	99.9		96.3	95.4	95.4	95.1	95.3	95.1	95.1	88.7	89.6	89.4	93.3
16	92.2	92.3	92.1	92.6	92.5	92.3	92.8	92.1	92.4	92.2	92.2	92.2	92.7	93.1	93.0		98.2	97.9	94.2	94.4	95.4	96.5	90.0	89.9	89.8	93.9
17	92.2	92.3	91.8	92.4	92.3	92.4	92.8	92.2	92.4	92.2	92.2	92.2	92.3	93.1	93.0	97.6		98.5	92.6	92.8	93.9	94.7	87.4	88.3	88.0	92.3
18	91.8	91.9	91.5	92.2	92.1	92.2	92.5	91.9	92.3	92.0	92.0	92.0	92.1	92.9	92.8	97.8	99.2		93.3	94.2	94.2	94.4	88.3	89.0	88.7	92.9
19	90.6	90.7	90.3	91.1	91.3	90.5	91.2	91.2	90.9	90.3	90.9	90.6	91.3	90.6	90.5	89.9	89.5	89.5		97.2	94.7	93.3	90.2	89.9	89.8	92.9
20	91.0	91.1	90.7	91.3	91.5	90.7	91.2	91.4	91.0	90.5	91.1	90.8	91.7	91.1	91.0	90.2	89.8	89.8	98.2		95.0	94.0	89.0	89.3	90.9	93.1
21	89.7	89.8	89.4	90.0	89.6	89.5	89.7	89.4	89.7	89.4	89.7	89.5	89.7	89.6	89.5	88.9	88.7	89.0	88.9	89.7		94.7	89.3	88.7	89.1	93.3
22	93.3	93.4	93.1	94.0	93.8	93.9	93.8	93.4	93.3	93.8	93.2	94.2	93.9	94.9	94.7	93.5	93.1	92.6	90.6	90.9	89.9		87.7	87.7	89.1	92.6
23	84.1	84.2	83.8	84.3	84.5	84.1	83.9	84.4	84.6	83.9	84.3	85.7	84.3	83.3	83.2	84.2	83.0	83.2	85.4	84.9	83.8	82.9		92.0	92.3	89.3
24	84.1	84.2	83.8	84.6	84.6	84.4	83.8	84.5	84.3	84.2	84.4	84.6	84.8	83.9	83.8	83.2	83.0	83.1	83.5	83.2	83.9	82.0	88.6		94.0	89.9
25	83.4	83.6	82.9	84.3	84.1	83.9	83.9	84.0	84.4	83.7	84.2	84.2	84.4	83.2	83.1	83.3	82.6	82.9	83.9	84.0	84.3	83.7	91.6	89.1		89.1
**Gottfried G4**	85.7	85.8	85.4	86.0	85.7	85.5	85.6	85.6	85.6	85.3	85.5	85.7	85.6	85.9	85.8	85.7	85.4	85.4	87.0	87.0	87.2	85.9	82.9	84.8	82.9	

Table S3. Nucleotide similarity (%) among RVA strains of the G9 genotype found in the current study and the prototype strain contained in the vaccine available in Spain (RVA/Pig-tc/USA/A2/198x/G9P97) (lower semi-matrix). Similarity among the amino acids coded by the same sequences is indicated in bold in the upper semi-matrix. Identity of RVA strains according to the nomenclature proposed by the RCWG is indicated in Table S10.

Nucleotides	**Amino acids**																						
Strain ID	**26**	**27**	**28**	**29**	**30**	**31**	**32**	**33**	**34**	**35**	**36**	**37**	**38**	**39**	**40**	**41**	**42**	**43**	**44**	**45**	**46**	**47**	**48**	**A2 G9**
26		***97.9***	***96.3***	***96.6***	***96.6***	***93.9***	***93.9***	***93.3***	***93.6***	***93.6***	***93.6***	***93.3***	***92.6***	***94.2***	***92.9***	***92.6***	***92.9***	***92.0***	***92.6***	***91.9***	***92.3***	***93.1***	***96.0***	***94.2***
27	98.2		***97.9***	***97.3***	***98.2***	***94.2***	***94.2***	***93.6***	***93.9***	***93.9***	***93.9***	***93.6***	***93.0***	***94.2***	***93.9***	***93.6***	***93.9***	***93.0***	***93.6***	***92.5***	***93.0***	***93.1***	***97.5***	***95.1***
28	95.4	98.2		***97.3***	***97.9***	***93.6***	***93.6***	***92.7***	***93.3***	***93.6***	***93.0***	***93.0***	***92.1***	***93.6***	***94.5***	***93.3***	***94.2***	***92.7***	***93.3***	***92.2***	***92.3***	***93.1***	***97.8***	***94.5***
29	95.2	96.0	95.5		***96.9***	***93.0***	***93.0***	***93.3***	***93.3***	***92.7***	***93.0***	***92.4***	***92.7***	***92.9***	***93.6***	***92.1***	***92.7***	***92.1***	***92.1***	***91.0***	***91.6***	***92.8***	***97.8***	***94.5***
30	94.9	95.9	95.4	94.6		***94.2***	***94.2***	***93.3***	***93.9***	***93.9***	***93.6***	***93.6***	***92.7***	***94.2***	***94.5***	***93.3***	***94.2***	***92.7***	***93.3***	***92.2***	***92.6***	***92.8***	***96.9***	***94.8***
31	93.3	93.7	93.9	93.2	93.1		***100.0***	***98.2***	***99.7***	***99.7***	***99.1***	***99.4***	***97.9***	***100.0***	***92.1***	***91.1***	***92.1***	***91.1***	***91.1***	***90.0***	***91.2***	***91.2***	***93.2***	***92.6***
32	92.8	93.3	93.4	92.9	92.6	99.5		***98.2***	***99.7***	***99.7***	***99.1***	***99.4***	***97.9***	***100.0***	***92.1***	***91.1***	***92.1***	***91.1***	***91.1***	***90.0***	***91.2***	***91.2***	***93.2***	***92.6***
33	92.7	93.1	93.1	92.8	92.6	98.4	98.3		***98.5***	***97.9***	***98.8***	***97.6***	***98.8***	***98.1***	***91.1***	***90.2***	***90.8***	***90.2***	***90.2***	***89.1***	***90.1***	***90.8***	***92.9***	***92.3***
34	92.7	93.1	93.3	93.2	92.5	99.4	99.3	98.4		***99.4***	***98.8***	***99.1***	***98.2***	***99.7***	***91.7***	***90.8***	***91.7***	***90.8***	***90.8***	***89.7***	***90.9***	***91.6***	***93.5***	***92.9***
35	92.7	93.1	93.3	92.8	92.5	99.2	99.5	98.0	99.0		***98.8***	***99.1***	***97.6***	***99.7***	***92.1***	***91.1***	***92.1***	***91.1***	***91.1***	***90.0***	***90.9***	***91.2***	***93.2***	***92.3***
36	92.6	93.0	93.1	92.7	92.4	99.2	99.1	98.8	99.0	98.8		***98.5***	***98.5***	***99.0***	***91.7***	***90.8***	***91.4***	***90.8***	***90.8***	***89.7***	***90.9***	***90.8***	***93.2***	***92.3***
37	92.5	92.9	93.1	92.6	92.3	99.2	99.1	98.0	99.4	98.8	98.8		***97.3***	***99.4***	***92.1***	***90.5***	***91.4***	***90.5***	***90.5***	***89.4***	***90.5***	***90.5***	***92.6***	***92.0***
38	92.4	92.8	92.8	92.3	92.6	98.3	98.2	98.8	98.3	97.9	98.6	97.9		***97.8***	***90.8***	***89.9***	***90.5***	***89.9***	***89.9***	***88.8***	***90.1***	***90.8***	***92.9***	***92.0***
39	92.6	92.9	93.1	92.7	92.4	99.4	99.3	98.2	99.6	98.9	98.9	99.4	98.0		***92.0***	***91.3***	***92.3***	***91.3***	***91.3***	***90.5***	***91.9***	***91.2***	***93.1***	***92.9***
40	90.9	91.5	91.3	90.8	91.1	89.8	89.5	89.3	89.6	89.4	89.3	89.6	89.5	91.9		***95.4***	***96.0***	***95.1***	***95.1***	***93.8***	***94.0***	***93.9***	***95.0***	***95.7***
41	90.9	91.3	90.9	89.7	90.3	90.2	89.7	89.8	89.8	89.6	89.5	89.5	89.5	89.7	91.9		***98.8***	***99.1***	***99.4***	***98.1***	***95.1***	***94.7***	***93.8***	***96.6***
42	90.8	91.4	90.8	89.8	90.8	90.5	90.2	90.3	90.3	90.1	90.2	90.1	90.2	90.2	92.4	98.1		***98.2***	***98.8***	***97.5***	***95.1***	***94.7***	***94.1***	***96.6***
43	90.5	91.1	90.4	89.7	90.0	89.8	89.5	89.4	89.4	89.4	89.3	89.2	89.2	89.3	91.9	97.2	96.8		***98.8***	***97.5***	***95.1***	***93.9***	***93.2***	***96.0***
44	90.2	90.6	90.2	89.3	89.4	89.7	89.6	89.1	89.5	89.5	89.4	89.5	89.0	89.4	91.7	94.8	94.9	94.8		***98.8***	***95.1***	***94.7***	***93.8***	***96.6***
45	90.6	90.9	90.5	89.4	89.8	89.6	89.3	89.0	89.3	89.2	89.1	89.0	88.9	89.2	91.0	94.9	94.9	95.4	95.5		***94.4***	***93.5***	***92.8***	***95.3***
46	89.7	90.1	89.7	89.2	89.8	89.2	88.7	88.5	89.1	88.7	88.9	88.7	88.6	89.1	90.6	91.6	91.7	91.3	90.9	90.0		***95.8***	***93.0***	***97.5***
47	91.5	91.9	90.8	90.6	91.2	89.9	90.1	90.3	90.5	89.8	89.7	89.8	90.2	90.1	91.4	91.5	92.0	91.0	90.3	90.9	92.6		***94.3***	***97.7***
48	94.9	95.8	96.4	96.1	94.6	93.5	93.0	92.4	93.3	92.9	92.8	93.1	92.4	93.1	91.0	90.7	90.3	89.9	90.1	90.3	89.3	90.7		***95.0***
A2 G9	92.5	93.3	92.8	92.2	92.7	92.0	91.6	91.9	92.0	91.5	91.4	91.5	91.6	91.6	94.1	93.7	93.5	93.2	92.3	92.6	95.0	96.8	92.4	

Table S4. Nucleotide similarity (%) among RVA strains of the G3 genotype found in the current study (lower semi-matrix). Similarity among the amino acids coded by the same sequences is indicated in bold in the upper semi-matrix. Identity of RVA strains according to the nomenclature proposed by the RCWG is indicated in Table S10.

Nucleotides					**Amino acids**							
Strain ID	**49**	**50**	**51**	**52**	**53**	**54**	**65**	**56**	**57**	**58**	**59**	**60**
49		***98.2***	***97.6***	***98.2***	***99.1***	***98.5***	***96.9***	***96.6***	***95.8***	***98.6***	***94.4***	***96.8***
50	96.7		***97.9***	***97.6***	***97.9***	***97.8***	***95.9***	***95.3***	***94.4***	***98.2***	***93.3***	***95.5***
51	97.0	96.9		***96.9***	***97.3***	***97.2***	***95.0***	***95.0***	***93.7***	***96.8***	***94.4***	***95.2***
52	97.9	97.0	97.4		***98.1***	***98.5***	***95.4***	***95.5***	***95.7***	***98.3***	***94.4***	***95.2***
53	97.5	96.8	97.4	97.6		***98.8***	***96.5***	***96.3***	***95.5***	***98.2***	***94.0***	***96.5***
54	97.6	96.9	97.4	97.9	98.3		***95.6***	***95.3***	***94.4***	***97.5***	***93.3***	***95.5***
55	95.6	94.2	94.4	95.0	95.1	95.2		***97.1***	***96.9***	***96.1***	***93.3***	***97.4***
56	94.2	92.9	93.4	93.7	93.8	93.7	93.9		***97.6***	***96.8***	***94.4***	***99.7***
57	94.5	93.2	93.4	94.1	94.0	93.6	94.4	95.5		***95.1***	***95.1***	***97.8***
58	94.9	94.2	94.5	94.5	94.8	94.6	92.9	92.7	92.2		***94.0***	***96.4***
59	87.3	85.3	87.0	86.8	86.9	86.5	85.8	86.2	86.6	85.7		***94.2***
60	94.7	93.2	93.7	94.0	94.2	94.1	94.2	99.6	95.8	92.8	86.2	

Table S5. Nucleotide similarity (%) among RVA strains of the G5 genotype found in the current study and the prototype strain contained in the vaccine available in Spain (RVA/Pig-tc/USA/LS00005_OSU/1975/G5P7) (lower semi-matrix). Similarity among the amino acids coded by the same sequences is indicated in bold in the upper semi-matrix. Identity of RVA strains according to the nomenclature proposed by the RCWG is indicated in Table S10.

Nucleotides			**Amino acids**		
Strain ID	**61**	**62**	**63**	**64**	**OSU G5**
61		***96.0***	***92.8***	***93.9***	***92.9***
62	92.6		***92.2***	***93.6***	***93.9***
63	84.9	84.2		***96.3***	***94.4***
64	85.9	85.4	94.0		***94.8***
OSU G5	85.6	84.9	86.7	88.0	

Table S6. Nucleotide similarity (%) among RVA strains of the G11 genotype found in the current study (lower semi-matrix). Similarity among the amino acids coded by the same sequences is indicated in bold in the upper semi-matrix. Identity of RVA strains according to the nomenclature proposed by the RCWG is indicated in Table S10.

Nucleotides	**Amino acids**	
Strain ID	**55**	**66**
65		***96***
66	95.5	

Table S7. Nucleotide similarity (%) among RVA strains of the P7 genotype found in the current study and the prototype strain contained in the vaccine available in Spain (RVA/Pig-tc/USA/LS00005_OSU/1975/G5P9[7] and RVA/Pig-tc/USA/A2/198x/G9P9[7]) (lower semi-matrix). Similarity among the amino acids coded by the same sequences is indicated in bold in the upper semi-matrix. Identity of RVA strains according to the nomenclature proposed by the RCWG is indicated in Table S10.

Nucleotides															**Amino acids**															
Strain ID	**26**	**62**	**44**	**63**	**53**	**7**	**42**	**2**	**11**	**5**	**54**	**3**	**28**	**8**	**1**	**13**	**9**	**10**	**6**	**12**	**4**	**16**	**30**	**34**	**59**	**52**	**46**	**27**	**OSU P[7]**	**A2 P[7]**
26		***100.0***	***97.5***	***94.0***	***94.6***	***98.2***	***97.9***	***97.9***	***96.8***	***97.9***	***97.5***	***97.5***	***97.1***	***98.2***	***97.8***	***97.9***	***97.5***	***96.1***	***95.7***	***95.7***	***97.1***	***95.7***	***94.6***	***95.3***	***93.6***	***93.6***	***89.4***	***93.6***	***93.9***	***87.7***
62	99.9		***97.5***	***94.0***	***94.6***	***98.2***	***97.9***	***97.9***	***96.8***	***97.9***	***97.5***	***97.5***	***97.1***	***98.2***	***97.8***	***97.9***	***97.5***	***96.1***	***95.7***	***95.7***	***97.1***	***95.7***	***94.6***	***95.3***	***93.6***	***93.6***	***89.4***	***93.6***	***93.9***	***87.7***
44	96.4	96.5		***93.2***	***95.0***	***97.1***	***96.8***	***96.8***	***95.7***	***96.8***	***96.4***	***96.4***	***96.1***	***97.1***	***96.8***	***96.8***	***96.4***	***95.0***	***94.6***	***94.6***	***96.0***	***95.7***	***94.6***	***95.7***	***93.6***	***93.6***	***91.2***	***94.0***	***94.3***	***87.4***
63	94.3	94.4	94.3		***91.7***	***94.7***	***94.3***	***94.3***	***93.2***	***94.3***	***94.0***	***94.0***	***94.3***	***94.2***	***94.3***	***94.3***	***94.7***	***92.8***	***92.8***	***92.8***	***93.6***	***96.6***	***92.8***	***92.8***	***92.1***	***92.1***	***89.4***	***91.1***	***91.7***	***84.8***
53	93.2	93.1	93.7	91.9		***95.3***	***95.7***	***95.0***	***94.6***	***95.0***	***94.6***	***94.6***	***95.0***	***95.2***	***95.0***	***95.0***	***95.3***	***93.2***	***93.6***	***93.6***	***95.0***	***93.2***	***93.2***	***94.3***	***93.2***	***93.2***	***89.8***	***92.4***	***93.6***	***87.0***
7	93.1	93.2	93.7	93.0	92.2		***99.6***	***99.6***	***98.6***	***99.6***	***99.3***	***99.3***	***98.9***	***99.6***	***99.6***	***99.6***	***99.3***	***97.1***	***97.5***	***97.5***	***98.6***	***95.7***	***95.0***	***95.3***	***94.3***	***94.3***	***90.3***	***94.0***	***95.3***	***88.9***
42	92.9	93.1	93.6	92.7	92.2	99.0		***99.3***	***98.2***	***99.3***	***98.9***	***98.9***	***99.3***	***99.3***	***99.3***	***99.3***	***99.6***	***96.8***	***97.1***	***97.1***	***98.9***	***95.3***	***95.3***	***95.7***	***94.6***	***94.6***	***90.7***	***94.4***	***95.7***	***89.3***
2	92.9	93.1	93.3	92.6	91.9	98.9	98.9		***98.2***	***99.3***	***99.6***	***99.6***	***98.6***	***99.3***	***100.0***	***99.3***	***98.9***	***96.8***	***97.1***	***97.1***	***98.2***	***95.3***	***94.6***	***95.0***	***93.9***	***93.9***	***90.7***	***94.4***	***95.7***	***89.3***
11	92.7	92.8	93.3	92.5	92.0	98.6	98.6	98.7		***98.2***	***97.9***	***98.6***	***97.5***	***98.2***	***98.2***	***98.2***	***97.9***	***97.9***	***98.2***	***98.2***	***97.1***	***95.0***	***93.6***	***93.9***	***92.8***	***92.8***	***88.9***	***92.4***	***93.9***	***87.4***
5	92.7	92.8	93.3	92.6	92.1	98.7	98.7	98.8	98.5		***98.9***	***98.9***	***98.6***	***100.0***	***99.3***	***100.0***	***98.9***	***96.8***	***97.1***	***97.1***	***98.2***	***95.3***	***94.6***	***95.0***	***94.3***	***93.9***	***90.3***	***93.6***	***95.0***	***88.5***
54	92.7	92.8	93.3	92.6	91.9	98.9	98.9	99.5	98.7	98.8		***99.3***	***98.2***	***98.9***	***99.6***	***98.9***	***98.6***	***96.4***	***96.8***	***96.8***	***97.8***	***95.0***	***94.3***	***94.6***	***93.6***	***93.6***	***90.7***	***94.0***	***95.7***	***89.3***
3	92.7	92.8	93.3	92.6	91.9	98.9	98.9	99.8	98.9	98.8	99.5		***98.2***	***98.9***	***99.6***	***98.9***	***98.6***	***96.4***	***96.8***	***96.8***	***97.8***	***95.0***	***94.3***	***94.6***	***93.6***	***93.6***	***90.7***	***94.0***	***95.3***	***88.9***
28	92.7	92.8	93.1	92.8	91.9	98.5	99.2	98.6	98.2	98.3	98.6	98.6		***98.5***	***98.6***	***98.6***	***99.6***	***96.8***	***97.1***	***97.1***	***98.2***	***95.3***	***95.3***	***95.7***	***94.6***	***94.6***	***90.3***	***94.8***	***95.7***	***89.3***
8	92.6	92.7	93.2	92.5	92.0	98.3	98.3	98.5	98.1	99.6	98.5	98.5	98.0		***99.3***	***100.0***	***98.9***	***96.7***	***97.1***	***97.1***	***98.2***	***95.6***	***94.8***	***95.2***	***94.1***	***93.7***	***90.3***	***93.6***	***94.8***	***88.5***
1	92.7	92.8	93.3	92.6	91.9	98.9	98.9	99.8	98.7	98.8	99.5	99.8	98.6	98.4		***99.3***	***98.9***	***96.8***	***97.1***	***97.1***	***98.2***	***95.3***	***94.6***	***95.0***	***93.9***	***93.9***	***90.7***	***94.4***	***95.7***	***89.3***
13	92.5	92.6	93.1	92.4	91.8	98.7	98.7	98.8	98.5	99.3	98.8	98.8	98.3	98.9	98.8		***98.9***	***96.7***	***97.1***	***97.1***	***98.2***	***95.3***	***94.6***	***95.0***	***94.3***	***93.9***	***90.3***	***93.6***	***95.0***	***88.5***
9	92.5	92.6	93.2	92.6	91.9	98.5	99.2	98.6	98.2	98.3	98.6	98.6	99.5	98.0	98.6	98.3		***96.4***	***96.8***	***96.8***	***98.6***	***95.7***	***95.7***	***96.1***	***95.0***	***95.0***	***90.7***	***94.8***	***96.1***	***89.7***
10	92.3	92.5	93.0	92.1	91.5	97.7	97.7	97.9	98.7	98.0	97.9	97.9	97.6	97.6	97.8	97.9	97.4		***99.6***	***99.6***	***95.7***	***94.3***	***92.5***	***92.8***	***92.5***	***92.5***	***88.4***	***91.6***	***92.8***	***86.2***
6	92.2	92.3	92.8	92.1	91.6	98.0	98.0	98.1	98.9	98.1	98.1	98.1	97.9	97.7	98.1	98.1	97.6	99.8		***100.0***	***96.0***	***93.9***	***92.5***	***92.8***	***92.5***	***92.5***	***88.4***	***91.6***	***92.8***	***86.2***
12	92.1	92.2	92.7	92.0	91.5	97.9	97.9	98.0	98.8	98.0	98.0	98.0	97.7	97.6	98.0	98.0	97.5	99.6	99.9		***96.0***	***93.9***	***92.5***	***92.8***	***92.5***	***92.5***	***88.4***	***91.6***	***92.8***	***86.2***
4	92.2	92.3	92.8	92.1	91.6	98.2	98.9	98.3	98.0	98.1	98.3	98.3	98.6	97.7	98.3	88.7	98.6	97.1	97.4	97.2		***94.6***	***94.6***	***95.0***	***93.9***	***93.9***	***89.8***	***93.6***	***94.6***	***88.1***
16	90.0	90.1	90.0	90.0	88.1	88.8	88.3	88.4	88.5	88.4	88.4	88.2	88.8	88.3	88.1	88.7	88.5	88.5	88.4	88.3	87.9		***96.8***	***95.7***	***95.0***	***95.0***	***88.9***	***94.8***	***93.6***	***86.6***
30	89.5	89.6	89.5	89.4	89.3	88.3	88.3	88.2	88.3	88.2	87.9	87.9	88.8	88.1	87.9	88.2	88.6	87.7	87.8	87.9	87.9	92.1		***97.1***	***96.8***	***96.8***	***91.7***	***95.6***	***95.3***	***89.3***
34	89.0	88.9	88.9	88.7	87.8	87.0	86.7	86.6	86.7	86.9	86.6	86.4	87.2	86.8	86.3	86.6	87.1	86.3	86.4	86.3	86.3	89.6	89.6		***96.1***	***96.1***	***91.2***	***98.0***	***95.3***	***88.5***
59	88.6	88.5	88.4	88.9	86.9	86.5	86.5	86.6	86.5	86.4	86.4	86.4	87.0	86.3	86.3	86.4	87.0	86.3	86.4	86.3	86.3	91.8	92.8	90.6		***98.6***	***91.2***	***94.0***	***94.6***	***87.4***
52	88.3	88.1	87.8	88.5	87.0	86.4	86.4	86.7	86.4	86.3	86.5	86.5	86.9	86.2	86.5	86.0	86.8	86.1	86.3	86.1	86.2	90.9	92.0	90.2	97.4		***90.3***	***94.0***	***94.6***	***87.7***
46	84.0	84.1	84.9	82.6	83.2	83.5	98.9	98.3	98.0	98.1	98.3	98.3	98.6	97.7	98.3	98.1	98.6	97.1	97.4	97.2	100.0	87.9	87.9	86.3	86.3	86.2		***89.4***	***93.1***	***86.6***
27	88.2	88.1	88.9	87.8	87.1	87.6	87.3	87.4	87.0	87.0	87.4	87.2	88.0	87.0	87.1	87.2	88.0	86.5	86.6	86.5	87.1	89.6	89.3	96.6	89.7	89.3	84.5		***94.0***	***87.5***
OSU P[7]	88.0	88.1	88.4	88.3	86.0	87.5	87.7	87.6	87.2	87.3	87.8	87.3	87.7	87.2	87.3	87.6	87.8	87.0	87.1	87.0	87.1	87.9	88.1	88.2	86.9	86.5	87.7	87.7		***90.4***
A2 P[7]	85.5	85.4	85.3	85.3	83.3	84.8	85.1	85.2	84.5	84.2	85.2	84.9	85.3	85.1	85.1	84.7	85.2	83.8	84.0	84.0	84.4	85.6	85.3	84.7	85.4	85.0	84.4	84.3	92.7	

Table S8. Nucleotide similarity (%) among RVA strains of the P23 genotype found in the current study (lower semi-matrix). Similarity among the amino acids coded by the same sequences is indicated in bold in the upper semi-matrix. Identity of RVA strains according to the nomenclature proposed by the RCWG is indicated in Table S10.

Nucleotides	Amino acids																					
Strain ID	**41**	**51**	**67**	**15**	**19**	**43**	**32**	**35**	**36**	**31**	**38**	**39**	**37**	**33**	**40**	**18**	**60**	**56**	**29**	**50**	**45**	**66**	**65**
41		***96.1***	***95.0***	***94.3***	***93.5***	***94.9***	***94.5***	***94.9***	***94.9***	***94.9***	***95.2***	***94.2***	***94.9***	***94.0***	***95.9***	***97.2***	***95.8***	***94.9***	***94.9***	***92.4***	***93.2***	***94.5***	***92.1***
51	94.9		***97.5***	***95.7***	***95.8***	***96.3***	***94.5***	***94.9***	***94.4***	***94.0***	***95.2***	***94.9***	***94.9***	***94.9***	***96.3***	***96.7***	***96.8***	***95.8***	***95.8***	***93.5***	***92.8***	***94.5***	***93.2***
67	93.3	96.1		***95.3***	***94.9***	***96.3***	***93.6***	***94.0***	***93.5***	***93.0***	***94.2***	***93.5***	***94.0***	***94.0***	***95.4***	***95.8***	***95.8***	***94.9***	***94.9***	***92.8***	***92.8***	***94.1***	***92.1***
15	93.3	95.3	94.7		***95.4***	***94.9***	***91.7***	***92.1***	***91.6***	***91.6***	***92.3***	***92.0***	***92.1***	***91.6***	***94.5***	***94.4***	***94.0***	***93.0***	***93.1***	***90.9***	***90.7***	***93.3***	***90.7***
19	92.7	94.4	93.5	96.1		***93.0***	***92.6***	***92.6***	***92.1***	***91.6***	***92.3***	***92.6***	***92.6***	***92.5***	***94.0***	***93.5***	***93.5***	***92.5***	***92.6***	***89.8***	***91.2***	***91.6***	***90.2***
43	93.0	94.9	95.2	95.2	94.1		***92.6***	***92.5***	***92.1***	***92.1***	***92.8***	***92.6***	***92.5***	***92.1***	***94.0***	***94.9***	***94.4***	***93.5***	***94.0***	***90.7***	***91.2***	***92.1***	***90.7***
32	87.6	86.7	87.0	87.0	87.8	87.2		***100.0***	***99.5***	***99.1***	***100.0***	***99.5***	***100.0***	***98.6***	***94.6***	***95.8***	***95.4***	***94.4***	***95.4***	***94.0***	***93.1***	***95.9***	***95.0***
35	87.6	86.7	87.0	87.0	88.1	87.1	99.7		***99.5***	***99.1***	***100.0***	***100.0***	***100.0***	***98.6***	***94.9***	***95.8***	***95.4***	***94.4***	***95.4***	***94.4***	***93.5***	***96.3***	***95.4***
36	86.9	85.7	86.0	86.0	86.8	86.1	98.6	98.6		***98.6***	***99.5***	***99.5***	***99.5***	***98.1***	***94.4***	***95.3***	***94.9***	***93.9***	***94.9***	***93.9***	***93.0***	***95.8***	***94.9***
31	87.3	86.1	86.4	86.4	87.1	86.5	99.1	99.1	98.3		***99.0***	***99.1***	***99.1***	***97.7***	***94.9***	***95.8***	***94.4***	***93.5***	***94.4***	***93.5***	***93.5***	***95.4***	***94.4***
38	87.0	86.1	86.7	86.7	87.2	86.4	99.2	99.2	98.1	98.6		***100.0***	***100.0***	***99.0***	***95.2***	***96.2***	***95.7***	***94.7***	***95.7***	***94.7***	***93.8***	***96.6***	***95.2***
39	87.1	86.7	87.2	87.2	87.5	86.8	99.1	99.4	98.3	98.8	98.9		***100.0***	***98.6***	***95.0***	***95.8***	***95.4***	***94.4***	***95.4***	***94.6***	***92.7***	***96.5***	***95.3***
37	87.3	86.4	87.0	87.0	87.8	86.8	99.4	99.4	98.3	98.8	98.9	99.4		***98.6***	***94.9***	***95.8***	***95.4***	***94.4***	***95.4***	***94.4***	***93.5***	***96.3***	***95.4***
33	87.4	86.5	86.8	86.8	87.6	87.0	99.2	99.2	98.4	98.9	98.9	98.9	98.9		***94.4***	***95.3***	***94.4***	***93.5***	***94.4***	***94.0***	***93.0***	***94.9***	***94.9***
40	87.6	89.4	88.8	88.8	89.8	88.8	92.5	92.6	92.2	92.6	92.0	92.3	92.3	92.7		***96.7***	***95.8***	***94.9***	***94.9***	***93.1***	***93.1***	***94.0***	***92.2***
18	88.7	88.9	88.4	88.4	88.6	88.8	91.3	91.3	91.0	91.3	90.7	91.0	91.0	91.2	94.1		***96.7***	***95.8***	***96.7***	***92.6***	***93.5***	***94.0***	***92.6***
60	87.5	88.0	87.2	87.2	87.6	87.3	91.2	91.2	90.7	90.9	90.9	91.5	91.5	91.0	93.1	92.7		***99.1***	***99.1***	***93.5***	***92.6***	***94.4***	***93.1***
56	87.3	87.8	87.0	87.0	87.4	87.1	90.8	90.9	90.3	90.5	90.5	91.2	91.1	90.7	92.7	92.4	99.7		***98.1***	***92.6***	***91.6***	***93.5***	***92.1***
29	87.5	88.0	87.2	87.2	87.6	87.3	91.2	91.2	90.7	90.9	90.9	91.5	91.5	91.0	93.1	93.0	99.1	98.8		***93.5***	***92.6***	***94.4***	***93.1***
50	84.5	84.9	83.9	83.9	83.6	82.6	85.8	85.8	85.2	85.1	85.3	85.9	85.4	85.6	85.3	84.9	84.6	84.2	84.6		***93.8***	***97.3***	***95.7***
45	85.4	85.5	84.3	84.3	85.2	84.2	86.4	86.4	85.5	86.1	85.6	86.2	86.1	86.2	85.6	87.0	85.5	85.1	85.5	85.1		***94.5***	***93.2***
66	85.9	86.4	85.5	85.5	85.6	85.1	86.2	86.6	85.5	85.9	85.7	86.7	86.2	86.1	86.1	86.2	85.7	85.3	85.3	85.3	94.3		***96.9***
65	85.5	86.1	84.8	84.8	85.0	84.3	87.3	87.3	86.3	86.7	86.4	87.4	87.0	87.1	86.4	85.8	85.3	85.0	85.3	85.0	94.3	94.9	

Table S9. Nucleotide similarity (%) among RVA strains of the P6 genotype found in the current study and the prototype strain contained in the vaccine available in Spain (RVA/Pig-tc/USA/LS00007_Gottfried/1975/G4P6) (lower semi-matrix). Similarity among the amino acids coded by the same sequences is indicated in bold in the upper semi-matrix. Identity of RVA strains according to the nomenclature proposed by the RCWG is indicated in Table S10.

					**Amino acids**						
Strain ID	**20**	**14**	**25**	**23**	**68**	**24**	**69**	**22**	**57**	**21**	**Gottfried P[6]**
20		***93.5***	***94.6***	***92.5***	***92.5***	***93.2***	***95.1***	***94.2***	***87.0***	***87.1***	***86.7***
14	90.5		***98.2***	***93.2***	***93.5***	***93.5***	***95.1***	***93.5***	***87.8***	***88.9***	***87.1***
25	89.9	97.7		***94.2***	***93.8***	***94.6***	***96.2***	***93.8***	***88.2***	***89.1***	***87.3***
23	90.2	90.2	89.7		***96.4***	***94.6***	***93.9***	***92.1***	***87.0***	***88.1***	***86.3***
68	90.7	91.6	90.4	95.2		***93.5***	***94.3***	***92.5***	***89.3***	***90.3***	***87.4***
24	90.3	91.3	91.1	91.7	91.6		***93.6***	***92.5***	***86.3***	***87.4***	***86.0***
69	89.6	90.8	90.0	91.0	92.6	90.8		***94.3***	***86.3***	***87.5***	***87.1***
22	89.5	91.0	90.5	89.8	91.4	89.6	91.3		***87.0***	***87.1***	***85.6***
57	85.1	85.2	84.6	85.1	86.4	84.1	83.6	84.4		***95.6***	***85.6***
21	84.8	85.3	84.6	85.7	86.3	84.3	83.7	83.6	96.2		***86.7***
Gottfried P[6]	83.3	82.9	82.5	82.7	82.4	82.5	82.7	81.2	81.0	81.1	

Table S10. Nucleotide similarity (%) among RVA strains of the P13 genotype found in the current study (lower semi-matrix). Similarity among the amino acids coded by the same sequences is indicated in bold in the upper semi-matrix. Identity of RVA strains according to the nomenclature proposed by the RCWG is indicated in Table S10.

Nucleotides			**Amino acids**			
Strain ID	**49**	**64**	**55**	**48**	**17**	**47**
49		***94.7***	***83.8***	***84.7***	***85.1***	***80.5***
64	93.4		***82.6***	***84.7***	***83.6***	***79.3***
55	85.4	85.0		***95.7***	***81.8***	***75.9***
48	85.4	84.8	94.9		***83.1***	***76.4***
17	81.4	81.6	80.9	79.0		***83.1***
47	79.1	78.8	76.7	76.2	80.9	

Table S11. Identity of RVA strains according to the nomenclature proposed by the Rotavirus Classification Working Group (RCWG)

Strain ID	code system RCWG	Strain ID	code system RCWG	Strain ID	code system RCWG
1	RVA/Pig-wt/ESP/Navarra107414/2020/G4P[7]	33	RVA/Pig-wt/ESP/Girona124968/2019/G9P[23]	65	RVA/Pig-wt/ESP/Orense135260.2/2020/G3P[23]
2	RVA/Pig-wt/ESP/Alava114818/2020/G4P[7]	34	RVA/Pig-wt/ESP/Barcelona117554/2020/G9P[7]	66	RVA/Pig-wt/ESP/Orense136923/2020/G11P[23]
3	RVA/Pig-wt/ESP/Alava115613/2020/G4P[7]	35	RVA/Pig-wt/ESP/Huesca113559/2020/G9P[23]	67	RVA/Pig-wt/ESP/Valencia120548/2020/GXP[23]
4	RVA/Pig-wt/ESP/Zaragoza120771/2019/G4P[7]	36	RVA/Pig-wt/ESP/Tarragona124577/2019/G9P[23]	68	RVA/Pig-wt/ESP/Malaga125288/2019/GxP[6]
5	RVA/Pig-wt/ESP/Barcelona121700/2019/G4P[7]	37	RVA/Pig-wt/ESP/Barcelona121707/2019/G9P[23]	69	RVA/Pig-wt/ESP/Almeria123770/2019/GxP[6]
6	RVA/Pig-wt/ESP/Huesca123408/2019/G4P[7]	38	RVA/Pig-wt/ESP/Lleida136467/2020/G9P[23]	A2G9	GenBank accession AB180971
7	RVA/Pig-wt/ESP/Zaragoza108260/2020/G4P[7]	39	RVA/Pig-wt/ESP/Lleida116188/2020/G9P[23]	A2P7	GenBank accession AB180977
8	RVA/Pig-wt/ESP/Zaragoza146764/2020/G4P[7]	40	RVA/Pig-wt/ESP/Girona107765/2020/G9P[23]	Gottfried G4	GenBank accession KR052772
9	RVA/Pig-wt/ESP/Zaragoza114059/2020/G4P[7]	41	RVA/Pig-wt/ESP/Zaragoza107115/2020/G9P[23]	Gottfried P6	GenBank accession KR052749
10	RVA/Pig-wt/ESP/Huesca126350/2019/G4P[7]	42	RVA/Pig-wt/ESP/Teruel115875/2020/G9P[7]	OSUG5	RVA/Pig-tc/USA/1975/OSU/G5P7 GenBank accession MT025939.1
11	RVA/Pig-wt/ESP/Huesca147560/2020/G4P[7]	43	RVA/Pig-wt/ESP/Barcelona146995/2020/G9P[23]	OSUP7	RVA/Pig-tc/USA/1975/OSU/G5P7 GenBank accession MT025935.1
12	RVA/Pig-wt/ESP/Huesca140386/2020/G4P[7]	44	RVA/Pig-wt/ESP/Alicante136655/2020/G9P[7]		
13	RVA/Pig-wt/ESP/Zaragoza120018/2019/G4P[7]	45	RVA/Pig-wt/ESP/Madrid129487/2019/G9P[23]		
14	RVA/Pig-wt/ESP/Avila111282/2020/G4P[6]	46	RVA/Pig-wt/ESP/Soria127003/2019/G9P[7]		
15	RVA/Pig-wt/ESP/Zaragoza111541/2020/G4P[23]	47	RVA/Pig-wt/ESP/Orense136924/2020/G9P[13]		
16	RVA/Pig-wt/ESP/Barcelona126745/2019/G4P[7]	48	RVA/Pig-wt/ESP/Zaragoza130763/2019/G9P[13]		
17	RVA/Pig-wt/ESP/Girona125546/2019/G4P[13]	49	RVA/Pig-wt/ESP/Toledo120391/2020/G3P[13]		
18	RVA/Pig-wt/ESP/Girona126230/2019/G4P[23]	50	RVA/Pig-wt/ESP/Madrid116607/2020/G3P[23]		
19	RVA/Pig-wt/ESP/Huesca122902/2019/G4P[23]	51	RVA/Pig-wt/ESP/Zaragoza107180/2020/G3P[23]		
20	RVA/Pig-wt/ESP/Cordoba113913/2020/G4P[6]	52	RVA/Pig-wt/ESP/Valladolid125688/2019/G3P[7]		
21	RVA/Pig-wt/ESP/Badajoz131861/2019/G4P[6]	53	RVA/Pig-wt/ESP/Teruel128484/2019/G3P[7]		
22	RVA/Pig-wt/ESP/Burgos125442/2019/G4P[6]	54	RVA/Pig-wt/ESP/Zaragoza120392/2019/G3P[7]		
23	RVA/Pig-wt/ESP/Badajoz114971/2020/G4P[6]	55	RVA/Pig-wt/ESP/Orense135260.1/2020/G11P[13]		
24	RVA/Pig-wt/ESP/Teruel128543/2019/G4P[6]	56	RVA/Pig-wt/ESP/Toledo143267/2020/G3P[23]		
25	RVA/Pig-wt/ESP/Badajoz126111/2019/G4P[6]	57	RVA/Pig-wt/ESP/Valladolid121140/2019/G3P[6]		
26	RVA/Pig-wt/ESP/Murcia106832/2020/G9P[7]	58	RVA/Pig-wt/ESP/Navarra126423/2019/G3P[x]		
27	RVA/Pig-wt/ESP/Granada135464/2020/G9P[7]	59	RVA/Pig-wt/ESP/Segovia122458/2019/G3P[7]		
28	RVA/Pig-wt/ESP/Zaragoza107413/2020/G9P[7]	60	RVA/Pig-wt/ESP/Valencia120195/2020/G3P[23]		
29	RVA/Pig-wt/ESP/Toledo121683/2019/G9P[23]	61	RVA/Pig-wt/ESP/Caceres119539/2020/G5P[X]		
30	RVA/Pig-wt/ESP/Toledo107854/2020/G9P[7]	62	RVA/Pig-wt/ESP/Almeria121891/2019/G5P[7]		
31	RVA/Pig-wt/ESP/Lleida110744/2020/G9P[23]	63	RVA/Pig-wt/ESP/Tarragona121102/2019/G5P[7]		
32	RVA/Pig-wt/ESP/Lleida107656/2020/G9P[23]	64	RVA/Pig-wt/ESP/Madrid123579/2019/G5P[13]		

Table S12. Amino acid residues defining neutralization domains of genotypes of the glycoprotein VP7. Amino acid mutations between Spanish pig strains from the present study and prototype vaccine strains Gottfried (KR052751), OSU (KR052772) and A2 (AB180971) (bold). The asterisks represent the same residues compared to any of the vaccine strains. Grey columns indicate conserved amino acids

	7-1a												7-1b								7-2								
	87	91	94	96	97	98	99	100	104	123	125	129	130	291	201	211	212	213	238	242	143	145	146	147	148	190	217	221	264
**Gottfried G4**	**S**	**T**	**N**	**N**	**E**	**W**	**K**	**D**	**Q**	**N**	**L**	**I**	**D**	**K**	**Q**	**N**	**T**	**A**	**D**	**T**	**R**	**A**	**S**	**G**	**E**	**S**	**T**	**S**	**G**
**OSU G5**	**N**	**T**	**A**	**T**	**K**	**W**	**T**	**E**	**Q**	**D**	**A**	**V**	**E**	**R**	**Q**	**D**	**I**	**N**	**D**	**S**	**K**	**D**	**G**	**N**	**L**	**S**	**T**	**A**	**G**
**A2 G9**	**T**	**T**	**G**	**T**	**E**	**W**	**K**	**N**	**Q**	**D**	**A**	**V**	**D**	**K**	**Q**	**N**	**K**	**A**	**D**	**N**	**K**	**D**	**S**	**T**	**L**	**S**	**E**	**N**	**G**
**G3**																													
RVA/Pig-wt/ESP/Toledo120391/2020/G3P[13]	A	R	*	*	S	*	*	*	*	*	*	*	*	*	*	*	*	*	S	*	*	*	A	A	*	*	*	*	*
RVA/Pig-wt/ESP/Madrid116607/2020/G3P[23]	A	R	*	*	S	*	*	*	*	*	*	*	*	*	*	*	*	*	S	*	*	*	A	A	S	*	*	*	*
RVA/Pig-wt/ESP/Valencia120195/2020/G3P[23]	*	*	*	*	S	*	*	*	*	*	*	*	*	*	*	*	*	*	S	*	*	*	A	A	*	*	*	*	*
RVA/Pig-wt/ESP/Zaragoza120392/2019/G3P[7]	A	R	*	*	S	*	*	*	*	*	*	*	*	*	*	*	*	*	S	*	*	*	A	A	*	*	*	*	*
RVA/Pig-wt/ESP/Valladolid121140/2019/G3P[6]	*	*	*	D	S	*	*	*	*	*	*	*	*	*	*	*	*	*	N	*	*	*	A	A	*	*	*	*	*
RVA/Pig-wt/ESP/Valladolid125688/2019/G3P[7]	A	R	*	K	S	*	*	*	*	*	*	*	*	*	*	*	*	*	N	*	*	*	A	A	*	*	*	*	*
RVA/Pig-wt/ESP/Navarra126423/2019/G3P[X]	A	R	*	*	S	*	*	*	*	*	*	*	*	n.a.	*	*	*	*	S	*	*	*	A	A	*	*	*	*	*
RVA/Pig-wt/ESP/Teruel128484/2019/G3P[7]	*	*	*	*	S	*	*	*	*	*	*	*	*	*	*	*	*	*	S	*	*	*	A	A	*	*	*	*	*
RVA/Pig-wt/ESP/Orense135260.2/2020/G3P[23]	*	*	*	*	S	*	*	*	*	*	*	*	*	*	*	*	*	*	S	*	*	*	A	A	*	*	*	*	*
RVA/Pig-wt/ESP/Toledo143267.2/2019/G3P[23]	*	*	*	*	S	*	*	*	*	*	*	*	*	*	*	*	*	*	S	*	*	*	A	A	*	*	*	*	*
RVA/Pig-wt/ESP/Segovia122458/2019/G3P[7]	*	R	*	D	S	*	*	*	*	*	*	*	*	n.a.	*	*	*	*	N	*	*	*	A	S	*	*	*	*	*
RVA/Pig-wt/ESP/Zaragoza107180/2020/G3P[23]	A	R	*	*	S	*	*	*	*	*	*	*	*	*	*	*	*	*	S	*	*	*	A	A	*	*	*	*	*
**G4**																													
RVA/Pig-wt/ESP/Navarra107414/2020/G4P[7]	A	I	*	D	*	*	*	*	*	*	*	*	*	*	*	*	*	*	N	*	*	V	*	*	*	*	*	*	*
RVA/Pig-wt/ESP/Zaragoza108260/2020/G4P[7]	A	*	*	D	*	*	*	*	*	*	*	*	*	*	*	*	*	*	N	*	*	I	*	*	*	*	*	*	*
RVA/Pig-wt/ESP/Avila111282/2020/G4P[6]	*	*	*	*	*	*	*	*	*	*	*	*	*	*	*	*	*	*	N	*	*	V	*	*	*	*	*	*	*
RVA/Pig-wt/ESP/Zaragoza111541/2020/G4P[23]	*	*	*	*	*	*	*	*	*	*	*	*	*	*	*	*	*	*	N	*	*	V	*	*	*	*	*	*	*
RVA/Pig-wt/ESP/Cordoba113913/2020/G4P[6]	*	*	S	*	*	*	*	*	*	*	*	*	*	*	*	*	*	*	*	*	*	*	*	*	*	L	*	R	*
RVA/Pig-wt/ESP/Salamanca114059/2020/G4P[7]	A	*	*	D	*	*	*	*	*	*	*	*	*	*	L	*	*	*	N	*	*	V	*	*	*	*	*	*	*
RVA/Pig-wt/ESP/Alava114818/2020/G4P[7]	A	I	*	D	*	*	*	*	*	*	*	*	*	*	*	*	*	*	N	*	*	V	*	*	*	*	*	*	*
RVA/Pig-wt/ESP/Badajoz114971/2020/G4P[6]	*	*	*	*	*	*	*	*	*	*	*	*	*	*	*	*	A	G	*	*	*	I	A	*	*	*	M	*	*
RVA/Pig-wt/ESP/Alava115613/2020/G4P[7]	A	I	*	D	*	*	*	*	*	*	*	*	*	*	*	*	*	*	N	*	*	*	*	*	*	*	*	*	*
RVA/Pig-wt/ESP/Huesca120086/2020/G4P[7]	*	*	*	*	*	*	*	*	*	*	*	*	*	*	*	*	*	*	*	*	*	*	*	*	*	*	*	*	*

RVA/Pig-wt/ESP/Zaragoza120018/2019/G4P[7]	A	*	*	D	*	*	*	*	*	*	*	*	*	*	*	*	*	*	*	*	*	V	*	*	*	*	*	*	*
RVA/Pig-wt/ESP/Zaragoza120771/2019/G4P[7]	A	*	*	D	*	*	*	*	*	*	*	*	*	*	*	*	*	*	*	*	*	I	*	*	*	*	*	*	*
RVA/Pig-wt/ESP/Huesca147560/2019/G4P[7]	A	*	*	D	*	*	*	*	*	*	*	*	*	*	*	*	*	*	N	*	*	I	*	*	*	*	*	*	*
RVA/Pig-wt/ESP/Huesca122902/2019/G4P[23]	*	*	*	*	*	*	*	*	*	*	*	*	*	*	*	*	A	*	*	*	*	T	*	*	*	*	*	R	*
RVA/Pig-wt/ESP/Huesca123408/2019/G4P[7]	A	*	*	Y	*	*	*	*	*	*	*	*	*	*	*	*	*	*	N	*	*	I	*	*	O	Q	*	*	*
RVA/Pig-wt/ESP/Barcelona121700/2019/G4P[7]	A	*	*	D	*	*	*	*	*	*	*	*	*	*	*	*	*	*	*	*	*	V	*	*	*	*	*	*	*
RVA/Pig-wt/ESP/Burgos125442/2019/G4P[6]	*	*	*	*	*	*	*	*	*	*	*	*	*	n.a.	*	*	*	*	*	*	*	*	*	*	Q	*	*	*	*
RVA/Pig-wt/ESP/Girona125546/2019/G4P[13]	A	*	*	*	*	*	*	*	*	*	*	*	*	*	*	*	*	*	N	*	*	*	*	*	*	*	*	*	*
RVA/Pig-wt/ESP/Badajoz126111/2019/G4P[6]	*	*	*	*	*	*	*	*	*	*	*	*	N	n.a.	*	*	*	G	*	*	*	I	A	*	*	L	M	*	*
RVA/Pig-wt/ESP/Girona126230/2019/G4P[23]	A	*	*	*	*	*	*	*	*	*	*	*	*	*	*	*	*	*	N	*	*	*	*	*	*	*	*	*	*
RVA/Pig-wt/ESP/Huesca126350/2019/G4P[7]	A	*	*	D	*	*	*	*	*	*	*	*	*	*	*	*	*	D	*	*	*	I	*	*	Q	*	*	*	*
RVA/Pig-wt/ESP/Barcelona126745/2019/G4P[7]	A	*	*	*	*	*	*	*	*	*	*	*	*	*	*	*	*	*	*	*	*	*	*	*	*	*	*	*	*
RVA/Pig-wt/ESP/Teruel128543/2019/G4P[6]	*	*	*	A	*	*	*	*	*	*	*	*	*	*	*	*	*	D	*	*	*	I	V	*	*	*	M	*	*
RVA/Pig-wt/ESP/Badajoz131861/2019/G4P[6]	*	*	S	*	*	*	*	*	*	*	*	*	N	*	*	*	*	*	*	*	*	*	*	*	*	*	*	*	*
RVA/Pig-wt/ESP/Spain140386/2020/G4P[7]	A	*	*	D	*	*	*	*	*	*	*	*	*	*	*	*	*	*	N	*	*	T	*	*	Q	*	*	*	*
RVA/Pig-wt/ESP/Zaragoza146764/2020/G4P[7]	A	*	*	D	*	*	*	*	*	*	*	*	*	*	*	*	*	*	*	*	*	V	*	*	*	*	*	*	*
**G5**																													
RVA/Pig-wt/ESP/Caceres119539/2020/G5P[X]	*	*	*	*	*	*	*	*	*	*	*	*	*	*	*	*	*	*	*	*	*	*	A	*	*	*	*	*	*
RVA/Pig-wt/ESP/Madrid123579/2019/G5P[13]	*	*	*	*	*	*	*	*	*	*	*	*	*	*	*	*	*	*	*	*	*	*	*	*	*	*	*	*	*
RVA/Pig-wt/ESP/Tarragona121102/2019/G5P[7]	*	*	*	D	*	*	*	*	*	*	*	*	*	*	*	*	*	*	*	*	*	*	*	*	*	*	L	*	*
RVA/Pig-wt/ESP/Almeria121891/2019/G5P[7]	*	*	*	*	*	*	*	*	*	*	*	*	*	*	*	*	*	G	*	*	*	*	A	*	*	*	*	*	*
**G9**																													
RVA/Pig-wt/ESP/Barcelona121707/2019/G9P[23]	*	A	R	A	*	*	*	*	*	*	*	*	N	*	*	*	*	*	*	*	*	*	*	R	*	*	*	E	*
RVA/Pig-wt/ESP/Toledo121683/2019/G9P[23]	*	N	E	*	*	*	*	*	*	*	*	*	*	*	*	*	P	T	*	D	*	*	*	*	*	*	*	R	*
RVA/Pig-wt/ESP/Murcia106832/2020/G9P[7]	*	N	*	P	*	*	*	*	*	*	*	*	*	*	*	*	*	*	*	D	*	*	*	*	*	*	*	R	*
RVA/Pig-wt/ESP/Zaragoza107115/2020/G9P[23]	*	*	*	*	*	*	*	*	*	*	*	*	*	*	*	*	*	*	*	*	*	N	*	*	*	*	*	*	*
RVA/Pig-wt/ESP/Zaragoza107413/2020/G9P[7]	*	N	R	*	*	*	*	*	*	*	*	*	*	*	*	*	P	*	*	D	*	*	*	*	*	*	*	R	*
RVA/Pig-wt/ESP/Lleida107656/2020/G9P[23]	*	A	R	A	*	*	*	*	*	*	*	*	N	*	*	*	*	*	*	*	*	*	*	R	*	*	*	E	*
RVA/Pig-wt/ESP/Girona107765/2020/G9P[23]	*	*	R	*	*	*	*	*	*	*	*	*	*	*	*	*	A	T	*	*	*	V	*	*	*	*	*	G	*
RVA/Pig-wt/ESP/Murcia107854/2020/G9P[7]	*	N	R	*	*	*	*	*	*	*	*	*	*	*	*	*	*	*	*	D	*	*	*	*	*	*	*	G	*
RVA/Pig-wt/ESP/Lleida110744/2020/G9P[23]	*	A	R	A	*	*	*	*	*	*	*	*	N	*	*	*	*	*	*	*	*	*	*	R	*	*	*	E	*

RVA/Pig-wt/ESP/Huesca113559/2020/G9P[23]	*	A	R	A	*	*	*	*	*	*	*	*	N	*	*	*	*	*	*	*	*	*	*	R	*	*	*	E	*
RVA/Pig-wt/ESP/Teruel115875/2020/G9P[7]	*	*	R	*	*	*	*	*	*	*	*	*	*	*	*	*	*	*	*	*	*	*	*	*	*	*	*	D	*
RVA/Pig-wt/ESP/Barcelona117554/2020/G9P[7]	*	A	R	A	*	*	*	*	*	*	*	*	N	*	*	*	*	*	*	*	*	*	*	R	*	*	*	E	*
RVA/Pig-wt/ESP/Lleida116188/2020/G9P[23]	*	A	R	A	*	*	*	*	*	*	*	*	N	*	*	*	*	*	*	*	*	*	*	R	*	*	*	E	*
RVA/Pig-wt/ESP/Tarragona124577/2019/G9P[23]	*	A	*	A	*	*	*	*	*	*	*	*	N	*	*	*	*	G	*	*	*	*	*	K	*	*	*	E	*
RVA/Pig-wt/ESP/Girona124968/2019/G9P[23]	*	A	*	A	*	*	*	*	*	*	*	*	N	*	*	*	*	G	*	*	*	*	*	*	*	*	*	E	*
RVA/Pig-wt/ESP/Madrid129487/2019/G9P[23]	*	*	*	*	*	*	*	*	*	*	*	*	*	*	*	*	*	*	*	*	*	*	*	*	*	*	*	*	*
RVA/Pig-wt/ESP/Zaragoza130763/2019/G9P[13]	*	N	*	*	*	*	*	*	*	*	*	*	*	*	*	*	P	T	*	D	*	*	*	*	*	*	*	R	*
RVA/Pig-wt/ESP/Granada135464/2020/G9P[7]	*	N	*	P	*	*	*	*	*	*	*	*	*	*	*	*	*	*	*	D	*	*	*	*	*	*	*	R	*
RVA/Pig-wt/ESP/Lleida136467/2020/G9P[23]	*	A	*	A	*	*	*	*	*	*	*	*	N	*	*	*	*	G	*	*	*	*	*	*	*	*	*	E	*
RVA/Pig-wt/ESP/Alicante136655/2019/G9P[7]	*	*	*	*	*	*	*	*	*	*	*	*	*	*	*	*	*	*	*	*	*	*	*	*	*	*	*	*	*
RVA/Pig-wt/ESPBarcelona146995/2020/G9P[23]	*	*	*	*	*	*	*	*	*	*	*	*	*	*	*	*	*	*	*	*	*	V	*	*	*	*	*	*	*
RVA/Pig-wt/ESP/Soria127003/2019/G9P[7]	A	*	*	*	*	*	*	*	*	*	*	*	*	D	*	*	*	*	*	*	*	*	Y	*	*	*	*	*	*
RVA/Pig-wt/ESP/Orense136924/2020/G9P[13]	*	*	*	*	*	*	*	*	*	*	*	*	*	D	*	*	*	*	*	*	*	*	Y	*	*	*	*	*	*
**G11**																													
RVA/Pig-wt/ESP/Spain136923/2020/G11P[23]	*	R	*	D	*	*	*	*	*	*	*	*	*	*	*	*	P	T	*	A	*	*	*	*	S	*	X	*	*
RVA/Pig-wt/ESP/Orense135260.1/2020/G11P[13]	*	R	*	D	*	*	*	*	*	*	*	*	*	*	*	*	P	T	N	*	*	*	*	*	S	*	*	*	*

n.a.: not available

Table S13. Amino acid residues defining neutralization domains of genotypes of the protein VP4. Amino acid mutations between Spanish pig strains from the present study and prototype vaccine strains Gottfried (KR052749), OSU (KR052770) and A2 (AB180977) (bold). The asterisks represent the same residues compared to any of the vaccine strains. Grey columns indicate conserved amino acids.

	8-1											8-2		8-3									8-4		
	100	146	148	150	188	190	192	193	194	195	196	180	183	113	114	115	116	125	131	132	133	135	87	88	89
**Gottfried P[6]**	**D**	**N**	**N**	**D**	**S**	**T**	**N**	**L**	**P**	**D**	**V**	**T**	**A**	**P**	**S**	**Q**	**D**	**V**	**E**	**N**	**S**	**D**	**I**	**N**	**K**
**OSU P[7]**	**D**	**T**	**T**	**S**	**Y**	**T**	**N**	**Y**	**D**	**T**	**V**	**T**	**A**	**Q**	**T**	**T**	**N**	**Q**	**E**	**N**	**T**	**Q**	**T**	**V**	**E**
**A2 P[7]**	**D**	**T**	**A**	**S**	**Y**	**A**	**D**	**Y**	**D**	**T**	**V**	**T**	**A**	**Q**	**A**	**T**	**N**	**Q**	**E**	**N**	**T**	**Q**	**T**	**V**	**E**
**P[6]**																									
RVA/Pig-wt/ESP/Cordoba113913/2020/G4P[6]	*	S	S	E	*	*	*	*	S	E	*	*	*	*	N	*	S	T	*	*	N	T	*	*	Q
RVA/Pig-wt/ESP/Avila111282/2020/G4P[6]	*	*	S	E	*	*	*	*	S	E	*	*	*	T	*	*	S	A	*	*	N	T	*	*	Q
RVA/Pig-wt/ESP/Badajoz114971/2020/G4P[6]	*	S	V	E	*	*	*	*	S	E	*	*	*	T	N	*	S	M	*	*	N	T	*	*	Q
RVA/Pig-wt/ESP/Valladolid121140/2019/G3P[6]	*	*	S	E	*	*	*	*	S	*	I	*	*	I	*	*	S	T	*	*	N	S	*	*	Q
RVA/Pig-wt/ESP/Teruel128543/2019/G4P[6]	*	S	S	E	*	*	*	*	S	E	*	*	*	T	N	*	S	A	*	*	N	T	*	*	Q
RVA/Pig-wt/ESP/Badajoz131861/2019/G4P[6]	*	*	S	E	*	*	*	*	*	N	I	*	*	I	*	*	S	T	*	*	N	S	*	*	Q
RVA/Pig-wt/ESP/Almeria123770/2019/GxP[6]	*	S	*	E	*	*	*	*	S	E	*	*	*	*	N	*	S	T	*	*	N	T	*	*	Q
RVA/Pig-wt/ESP/Malaga125288/2019/GxP[6]	*	*	S	E	*	*	*	*	S	E	I	*	*	T	N	*	S	T	*	*	N	T	*	*	Q
RVA/Pig-wt/ESP/Burgos125442/2019/G4P[6]	*	*	*	E	*	*	*	*	F	E	*	*	*	T	N	*	S	T	*	*	N	T	*	*	Q
RVA/Pig-wt/ESP/Badajoz126111/2019/G4P[6]	*	S	S	E	*	*	*	*	S	E	*	*	*	T	*	*	S	A	*	*	N	T	*	*	Q
**P[7]**																									
RVA/Pig-wt/ESP/Zaragoza120018.1/2019/G4P[7]	*	*	*	*	*	*	*	*	*	*	*	*	V	*	*	*	*	*	*	*	*	*	*	*	*
RVA/Pig-wt/ESP/Zaragoza120392/2019/G3P[7]	*	*	*	*	*	*	*	*	*	*	*	*	V	*	*	*	*	*	*	*	*	*	*	*	*
RVA/Pig-wt/ESP/Soria127003/2019/G9P[7]	*	*	*	*	*	*	*	*	*	*	*	*	*	*	*	*	*	*	*	*	V	H	*	*	*
RVA/Pig-wt/ESP/Zaragoza120771/2019/G4P[7]	*	*	*	*	*	*	*	*	*	*	*	*	V	*	*	*	*	*	*	*	*	*	A	A	*
RVA/Pig-wt/ESP/Murcia106832/2020/G9P[7]	*	*	*	*	*	*	*	*	*	*	*	*	V	*	*	*	*	*	*	*	*	*	*	*	*
RVA/Pig-wt/ESP/Zaragoza107413/2020/G9P[7]	*	*	*	*	*	*	*	*	*	*	*	*	V	*	*	*	*	*	*	*	*	*	*	*	*
RVA/Pig-wt/ESP/Navarra107414/2020/G4P[7]	*	*	*	*	*	*	*	*	*	*	*	*	V	*	*	*	*	*	*	*	*	*	*	*	*
RVA/Pig-wt/ESP/Toledo107854/2020/G9P[7]	*	*	*	N	*	*	*	*	*	*	*	*	*	*	*	*	T	*	*	*	*	*	*	*	*
RVA/Pig-wt/ESP/Zaragoza108260/2020/G4P[7]	*	*	*	*	*	*	*	*	*	*	*	*	V	*	*	*	*	*	*	*	*	*	*	*	*
RVA/Pig-wt/ESP/Zaragoza114059/2020/G4P[7]	*	*	*	*	*	*	*	*	*	*	*	*	V	*	*	*	*	*	*	*	*	*	*	*	*
RVA/Pig-wt/ESP/Alava114818/2020/G4P[7]	*	*	*	*	*	*	*	*	*	*	*	*	V	*	*	*	*	*	*	*	*	*	*	*	*
RVA/Pig-wt/ESP/Alava115613/2020/G4P[7]	*	*	*	*	*	*	*	*	*	*	*	*	V	*	*	*	*	*	*	*	*	*	*	*	*
RVA/Pig-wt/ESP/Teruel115875/2020/G9P[7]	*	*	*	*	*	*	*	*	*	*	*	*	V	*	*	*	*	*	*	*	*	*	*	*	*
RVA/Pig-wt/ESP/Barcelona117554/2020/G9P[7]	*	*	*	*	*	*	*	*	*	*	*	*	*	*	*	*	T	*	*	*	*	*	*	*	*

RVA/Pig-wt/ESP/Barcelona121700/2019/G4P[7]	*	*	*	*	*	*	*	*	*	*	*	*	V	*	*	*	*	*	*	*	*	*	*	*	*
RVA/Pig-wt/ESP/Almeria121891/2019/G5P[7]	*	*	*	*	*	*	*	*	*	*	*	*	V	*	*	*	*	*	*	*	*	*	*	*	*
RVA/Pig-wt/ESP/Segovia122458/2019/G3P[7]	*	*	*	*	*	*	*	*	*	*	*	*	*	*	*	*	T	*	*	*	*	*	*	*	*
RVA/Pig-wt/ESP/Huesca147560/2020/G4P[7]	*	*	*	*	*	*	*	*	*	*	*	*	V	*	*	*	*	*	*	*	*	*	*	*	*
RVA/Pig-wt/ESP/Zaragoza146764/2020/G4P[7]	*	*	*	*	*	*	*	*	*	*	*	*	V	*	*	*	*	*	*	*	*	*	*	*	*
RVA/Pig-wt/ESP/Huesca140386/2020/G4P[7]	*	*	*	*	*	*	*	*	*	*	*	*	V	*	*	*	*	*	*	*	*	*	*	*	*
RVA/Pig-wt/ESP/Alicante136655/2020/G9P[7]	*	*	*	*	*	*	*	*	*	A	*	*	*	*	*	*	*	*	*	*	A	*	*	*	*
RVA/Pig-wt/ESP/Granada135464/2020/G9P[7]	*	*	*	*	*	*	*	*	*	*	*	*	*	*	*	*	T	*	*	*	*	*	*	*	*
RVA/Pig-wt/ESP/Huesca123408/2019/G4P[7]	*	*	*	*	*	*	*	*	*	*	*	*	V	*	*	*	*	*	*	*	*	*	*	*	*
RVA/Pig-wt/ESP/Valladolid125688/2019/G3P[7]	*	*	*	*	*	*	*	*	*	*	*	*	*	*	*	*	T	*	*	*	*	*	*	*	*
RVA/Pig-wt/ESP/Huesca126350/2019/G4P[7]	*	*	*	*	*	*	*	*	*	*	*	*	V	*	*	*	*	*	*	*	*	*	*	*	*
RVA/Pig-wt/ESP/Barcelona126745/2019/G4P[7]	*	*	*	*	*	*	*	*	*	*	*	*	*	*	*	*	T	R	*	*	*	*	*	*	*
RVA/Pig-wt/ESP/Teruel128484/2019/G3P[7]	*	*	*	*	*	*	*	*	*	*	*	*	*	*	*	*	*	*	*	*	*	*	*	*	A
RVA/Pig-wt/ESP/Tarragona121102/2019/G5P[7]	*	*	*	*	*	*	*	*	*	*	*	*	*	*	*	*	*	*	*	*	*	L	*	*	
**P[13]**																									
RVA/Pig-wt/ESP/Toledo120391/2020/G3P[13]	*	S	*	T	*	*	*	*	S	A	I	*	*	*	R	E	L	R	*	*	A	*	N	D	*
RVA/Pig-wt/ESP/Orense136924/2020/G9P[13]	S	S	*	*	*	M	*	*	*	M	I	*	*	S	Q	E	L	*	*	*	*	*	S	*	*
RVA/Pig-wt/ESP/Orense135260.1/2020/G11P[13]	*	*	H	T	*	*	*	*	S	A	I	*	*	*	Q	E	T	*	*	*	V	*	N	D	*
RVA/Pig-wt/ESP/Zaragoza130763/2019/G9P[13]	*	S	H	T	*	*	*	*	S	A	I	*	*	*	Q	E	T	*	*	*	V	*	N	D	*
RVA/Pig-wt/ESP/Madrid123579/2019/G5P[13]	*	S	*	T	*	*	*	*	S	A	I	*	*	*	R	E	L	R	*	*	*	*	N	D	*
RVA/Pig-wt/ESP/Girona125546/2019/G4P[13]	*	S	*	T	*	*	S	*	N	*	I	*	*	S	Q	E	V	R	*	*	*	*	D	D	*
**P[23]**																									
RVA/Pig-wt/ESP/Zaragoza107115/2020/G9P[23]	*	D	*	T	*	*	*	*	*	*	*	*	*	T	*	E	*	*	*	*	V	T	S	*	A
RVA/Pig-wt/ESP/Zaragoza107180/2020/G3P[23]	*	D	*	T	*	*	*	*	*	*	*	*	*	T	*	E	S	*	*	*	V	T	S	T	A
RVA/Pig-wt/ESP/Lleida107656/2020/G9P[23]	*	D	*	T	*	*	*	*	*	*	*	*	V	T	*	*	S	P	*	*	V	T	S	*	A
RVA/Pig-wt/ESP/Girona107765/2020/G9P[23]	*	D	*	T	*	*	S	*	*	*	*	*	*	T	*	*	S	*	*	*	V	T	S	*	A
RVA/Pig-wt/ESP/Lleida110744/2020/G9P[23]	*	D	*	T	*	*	*	*	*	*	*	*	V	T	*	*	S	P	*	*	V	T	S	*	A
RVA/Pig-wt/ESP/Zaragoza111541/2020/G4P[23]	*	D	*	T	*	*	*	*	*	*	*	*	*	A	*	E	S	*	*	*	V	T	S	T	A
RVA/Pig-wt/ESP/Huesca113559/2020/G9P[23]	*	D	*	T	*	*	*	*	*	*	*	*	V	T	*	*	S	P	*	*	V	T	S	*	A
RVA/Pig-wt/ESP/Lleida116188/2020/G9P[23]	*	D	*	T	*	*	*	*	*	*	*	*	V	T	*	*	S	P	*	*	V	T	S	*	A
RVA/Pig-wt/ESP/Madrid116607/2020/G3P[23]	*	D	*	T	*	*	*	*	*	*	*	*	*	T	*	*	S	*	*	*	V	T	S	*	A
RVA/Pig-wt/ESP/Valencia120195/2020/G3P[23]	*	D	*	T	*	*	*	*	*	*	*	*	*	T	*	*	S	*	*	*	I	T	S	*	A

RVA/Pig-wt/ESP/Valencia120548/2020/GXP[23]	*	D	*	T	*	*	*	*	*	*	*	*	*	T	*	E	S	*	*	*	V	T	S	T	A
RVA/Pig-wt/ESP/Toledo121683/2019/G9P[23]	*	D	*	T	*	*	*	*	*	*	*	*	V	T	*	*	S	*	*	*	I	T	S	*	A
RVA/Pig-wt/ESP/Barcelona121707/2019/G9P[23]	*	D	*	T	*	*	*	*	*	*	*	*	V	T	*	*	S	P	*	*	V	T	S	*	A
RVA/Pig-wt/ESP/Huesca122902/2019/G4P[23]	*	D	*	T	*	*	*	*	*	*	*	*	*	A	*	E	S	*	*	*	V	T	S	T	A
RVA/Pig-wt/ESP/Barcelona146995/2020/G9P[23]	*	D	*	T	*	*	*	*	*	*	*	*	*	T	*	E	S	*	*	*	V	T	S	T	A
RVA/Pig-wt/ESP/Toledo143267.2/2020/G3P[23]	*	D	*	T	*	*	*	*	*	*	*	*	*	T	*	*	S	*	*	*	I	T	S	*	A
RVA/Pig-wt/ESP/Orense136923/2020/G11P[23]	*	D	*	T	*	*	*	*	*	*	*	*	*	A	*	*	S	*	*	*	V	T	S	*	A
RVA/Pig-wt/ESP/Lleida136467/2020/G9P[23]	*	D	*	T	*	*	*	*	*	*	*	*	V	T	*	*	S	P	*	*	V	T	S	*	A
RVA/Pig-wt/ESP/Orense135260.2/2020/G3P[23]	*	D	*	T	*	*	*	*	*	*	*	*	*	T	L	*	S	*	*	*	V	M	S	*	A
RVA/Pig-wt/ESP/Madrid129487/2019/G9P[23]	*	D	*	T	*	*	*	*	*	*	*	*	*	T	*	*	S	*	*	*	V	T	S	*	A
RVA/Pig-wt/ESP/Tarragona124577/2019/G9P[23]	*	D	*	T	*	*	*	*	*	*	*	*	V	T	*	*	S	P	*	*	V	T	S	*	A
RVA/Pig-wt/ESP/Girona124968/2019/G9P[23]	*	D	*	T	*	*	*	*	*	*	*	*	V	T	*	*	S	P	*	*	V	T	S	*	A
RVA/Pig-wt/ESP/Girona126230/2019/G4P[23]	*	D	*	T	*	*	*	*	*	*	*	*	V	T	*	*	S	*	*	*	V	T	S	*	A
